# Chætognath transcriptome reveals ancestral and unique features among bilaterians

**DOI:** 10.1186/gb-2008-9-6-r94

**Published:** 2008-06-04

**Authors:** Ferdinand Marlétaz, André Gilles, Xavier Caubit, Yvan Perez, Carole Dossat, Sylvie Samain, Gabor Gyapay, Patrick Wincker, Yannick Le Parco

**Affiliations:** 1 CNRS UMR 6540 DIMAR, Station Marine d'Endoume, Centre d'Océanologie de Marseille, Chemin de la Batterie des Lions, 13007, Marseille, France; 2 Université de la Méditerranée Aix-Marseille II, Bd Charles Livon, 13284, Marseille, France; 3 Université de Provence Aix-Marseille I, place Victor-Hugo, 13331, Marseille, France; 4 CNRS UMR 6116 IMEP, Centre St Charles, place Victor-Hugo, 13331, Marseille, France; 5 CNRS UMR 6216, IBDML, Campus de Luminy, Route Léon Lachamp, 13288, Marseille, France; 6 Genoscope (CEA), rue Gaston Crémieux, BP5706, 91057 Evry, France; 7 CNRS, UMR 8030, rue Gaston Crémieux, BP5706, 91057 Evry, France; 8 Université d'Evry, Boulevard François Mitterrand, 91025, Evry, France

## Abstract

**Background:**

The chætognaths (arrow worms) have puzzled zoologists for years because of their astonishing morphological and developmental characteristics. Despite their deuterostome-like development, phylogenomic studies recently positioned the chætognath phylum in protostomes, most likely in an early branching. This key phylogenetic position and the peculiar characteristics of chætognaths prompted further investigation of their genomic features.

**Results:**

Transcriptomic and genomic data were collected from the chætognath *Spadella cephaloptera *through the sequencing of expressed sequence tags and genomic bacterial artificial chromosome clones. Transcript comparisons at various taxonomic scales emphasized the conservation of a core gene set and phylogenomic analysis confirmed the basal position of chætognaths among protostomes. A detailed survey of transcript diversity and individual genotyping revealed a past genome duplication event in the chætognath lineage, which was, surprisingly, followed by a high retention rate of duplicated genes. Moreover, striking genetic heterogeneity was detected within the sampled population at the nuclear and mitochondrial levels but cannot be explained by cryptic speciation. Finally, we found evidence for trans-splicing maturation of transcripts through splice-leader addition in the chætognath phylum and we further report that this processing is associated with operonic transcription.

**Conclusion:**

These findings reveal both shared ancestral and unique derived characteristics of the chætognath genome, which suggests that this genome is likely the product of a very original evolutionary history. These features promote chætognaths as a pivotal model for comparative genomics, which could provide new clues for the investigation of the evolution of animal genomes.

## Abstract 2

The chætognath transcriptome reveals unusual genomic features in the evolution of this protostome and suggests that it could be used as a model organism for bilaterians.

## Background

The recent shift of genomic biology from conventional model organisms to evolutionarily relevant species has led to the questioning of numerous ideas about metazoan evolution. For instance, the recently released genome of the starlet anemone has revealed a striking conservation with its vertebrate counterparts despite an apparent morphological gap between these organisms [1]. On the contrary, whereas the Hox gene clusters have been considered for a long time as structures strictly required for the development of the common bilaterian body plan, they were found to be disorganized or even dislocated in animals such as nematodes or urochordates [2,3]. These cases illustrate the interest of genomic insights from organisms that display either peculiar morphological characteristics or have key phylogenetic positions.

Interestingly, chætognaths, also known as arrow worms, fulfill both of these criteria: they have one of the most intriguing sets of morphological and developmental characteristics among animals and their phylogenetic position was recently reevaluated as a pivotal one for the understanding of animal evolution [4]. These free-living marine creatures represent one of the major predators of the zooplancton food-chain but the phylum is mainly known for its original mosaic of morphological characteristics that have puzzled zoologists for years [5]. Their nervous system exhibits typical protostome features, such as ventral nervous mid-body ganglions and circum-esophageal fibers [6], whereas the enterocoelous formation of their body cavity and the secondary emergence of their mouth are embryological features traditionally related to deuterostomes [7]. Strikingly, this original body plan has been conserved since the lowermost Cambrian period as shown by convincing fossil evidence [8,9]. First attempts to position chætognaths using molecular phylogeny were difficult because small subunits (SSUs) and large subunits (LSUs) of ribosomal RNA genes display very fast evolutionary rates that hinder accurate tree reconstruction [10-12]. Subsequent analysis of their mitochondrial genome prompted classification of chætognaths among protostomes, but their exact branching in this clade remains elusive [13,14]. The Hox genes of chætognaths are distinct from those typical of other protostomes: their original *MedPost *gene shares similarity with both median and posterior classes [15] and the posterior Hox genes that were recently identified in these animals are neither related to the AbdB nor Post1/2 classes, which are specific for ecdysozoans and lophotrochozoans, respectively [16].

Recently, the phylogenomic approach has provided the opportunity to sum up the phylogenetic signal from hundreds of genes and thereby to increase the resolution of the phylogenies [17]. Two different phylogenomic studies involving different chætognath species and based on different samples of nuclear genes have assessed the phylogenetic position of chætognaths. They have both provided strong support for the inclusion of chætognaths within protostomes [17-19]. Matus *et al. *[19] suggested the branching of chætognaths at the base of lophotrochozoans on the basis of 72 nuclear genes described as valuable phylogenetic markers by Philippe *et al*. [20]. Conversely, using a slightly larger taxonomic sampling and 78 ribosomal protein (RP) genes, Marlétaz *et al. *[18] proposed that chætognaths are the sister group of all other protostomes. This last hypothesis has deep implications for the evolution of developmental patterns among bilaterians since it promotes the view that deuterostome-like developmental features such as enterocoely or a secondary mouth opening may be ancestral among bilaterians. Interestingly, recent insights into the structure of the nervous system of chætognaths suggest that these organisms have an intra-epidermal non-centralized nerve plexus, such as those observed in hemichordates or cnidarians [6]. This is another example of a putative ancestral characteristic in this phylum. Then, both the phylogenetic position of chætognaths and their peculiar morphology and development indicate that these organisms are pivotal for the understanding of animal evolution.

The expressed sequence tag (EST) approach provides an interesting opportunity to survey genomes and to perform comparisons between organisms. For instance, whole transcriptome comparisons based on ESTs initially suggested that the gene repertory shared by all metazoans is larger than expected [21]. Moreover, in regard to the unexpected genetic complexity of cnidarians, the evolutionary extent of gene losses observed in nematodes and *Drosophila *remains to be defined [21]. Through their original phylogenetic position, chætognaths offer the opportunity to check whether the ancestral protostome transcriptome has already undergone such gene losses or remains close to the ancestral bilaterian gene set conserved between vertebrates and cnidarians. Furthermore, the identification of a core set of metazoan conserved genes from a large range of organisms provides marker genes for phylogenomic analyses and signature genes as rare genomic changes, which could lead to a reevaluation of animal phylogeny [22,23].

Here, we describe an overview of *Spadella cephaloptera *genomics through fine-scale mining of consistent transcriptomic data. Although the morphology of chætognaths has been extensively described, only a few molecular studies have focused on these strange organisms. The transcriptome of chætognaths reveals a strong similarity with that of other bilaterians. This comparative framework allowed detection of molecular signatures and stressed the usefulness of RPs as marker genes for phylogenomic reconstruction. Along with the structural RNAs, RPs are major components of the ribosome translation complex [24]. They constitute a set of remarkably conserved genes among eukaryotes, which have not been significantly affected by lineage-specific duplication [25]. We took advantage of their high levels of expression, which allowed the assembly of a large dataset with extensive taxon sampling using ESTs. We then investigated the origin of the polymorphisms observed within the EST collection in the light of genome duplication or cryptic speciation as alternative explanatory hypotheses. Lastly, we found evidence for trans-splicing mRNA maturation in chætognaths from this EST data. This original mRNA processing mechanism involves the addition of a spliced-leader sequence at the 5' extremity of transcripts. This mechanism has been discovered in several animal phyla by analyzing other EST collections [26]. Interestingly, the occurrence of trans-splicing in chætognaths has deep implications for the evolutionary origin and functional significance of this mechanism.

## Results and discussion

### Partial transcriptome of the chætognath *S. cephaloptera*

**Figure 1:**
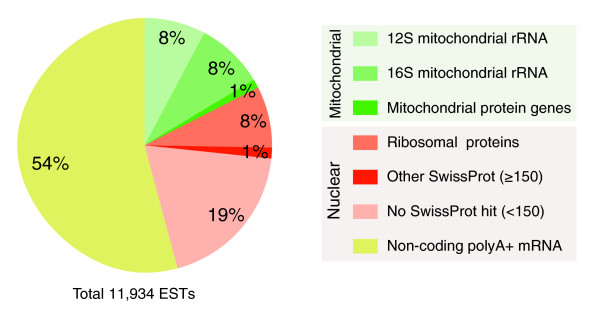
Overall composition of the EST collection. The annotation of transcripts is based on SwissProt (score >150) and led to identification of mitochondrial genes. The conceptual translation of ESTs allowed detection of those that include coding sequences. The large portion of non-coding polyadenylate nuclear transcripts and RPs among nuclear transcripts is the most prominent aspect of this distribution as well as the unexpected presence of mitochondrial rRNAs (12 and 16S) related to their polyadenine stretches.

The sequencing of an EST collection of the juvenile-staged chætognath *S. cephaloptera *offered the opportunity to explore the transcriptome of this evolutionarily significant organism. The survey of sequence length and quality supported the accuracy of these data (Figure S1 in Additional data file 1). During these steps, we noticed that 16% of sequences match mitochondrial rRNA sequences (12S and 16S rRNAs, Figure 1) probably because the long polyadenine stretches of these rRNA molecules were isolated by the oligos-dT employed for mRNA isolation (see Materials and methods). We attempted to build clusters that gathered all transcripts from a unique gene so as to deal with a non-redundant partial transcriptome. However, the low complexity regions of some ESTs, which did not include an accurate open reading frame, hindered this process. Thus, ESTs were sorted into predicted coding and non-coding sequences using conceptual translation, and the coding transcripts were retained for comparative analyses. The overall content of the EST collection was evaluated using these steps (Figure 1). We noticed that up to 54% of the ESTs could be non-coding polyadenylated RNA, a striking figure that is, however, similar to that obtained for the human genome [27]. The removal of non-coding sequences greatly improved clustering efficiency, yielding 1,447 clusters, of which 459 include more than one sequence (Figure S1 in Additional data file 1). A total of 694 of these clusters have significant matches within a protein database (TrEMBL, score >50) and 250 have clear homologs in this database with an average of 72% identity (score >150). Among the transcripts that match nuclear coding genes, the RP genes are largely represented compared to other genes similar to SwissProt entries (Figure 1).

The average gene content of the library was checked regarding functional annotation as implemented in Gene Ontology [28]. The *S. cephaloptera *library exhibited a broad diversity of functional classes with a majority of transcripts involved in metabolism or cellular activities and a non-negligible amount of transcripts involved in development (Figure S2 in Additional data file 1), which is consistent with the juvenile stage of the animals used. Hence, this EST collection contains representative, high quality sequences, providing suitable material for comparative analyses.

### Gene core conservation

**Figure 2:**
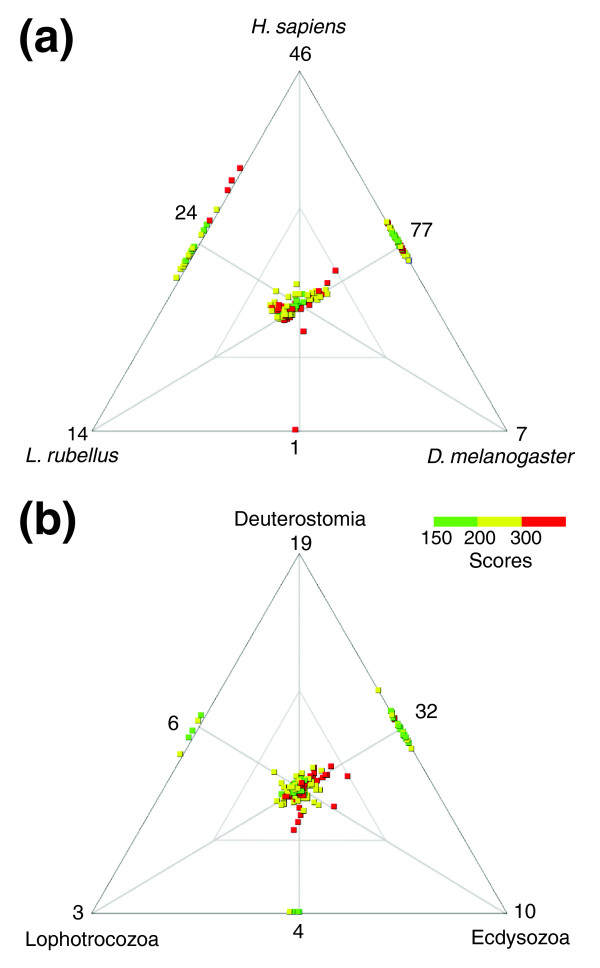
Visualization of relative similarity between the transcriptome of *S. cephaloptera *and **(a) **selected species or **(b) **corresponding clades: *H. sapiens *as a deuterostome, *D. melanogaster *as an ecdsyzoan and *L. rubellus *as a lophotrochozoan. The graphs are based on whole transcriptome Blast comparisons and the plotting of respective Blast scores was performed using Simitri [77] (cut-off score 150). Genes at the center of the plot are equally related to the three databases and hence represent valuable phylogenetic markers, whereas genes attracted by a node share a greater similarity with the corresponding database. Genes on the edge do not have a match in the database from the opposite vertex and those on the vertex only have a match in the corresponding database; these two types of genes constitute candidates for signature genes that have possibly been lost in a peculiar lineage. The color scale indicates the relevancy of scores.

The set of non-redundant chætognath transcripts was compared with several databases using the Blast program. These databases first included sets of transcripts of representative species belonging to the most important clades of bilaterians: *Drosophila melanogaster *as an ecdysozoan, *Lumbricus terrestris *as a lophotrochozoan and *Homo sapiens *as a deuterostome. These comparisons were depicted through the plotting of respective similarity scores for all transcripts that have a significant match to at least one of these species (score >150, Figure 2). This comparison demonstrated that a pool of 141 transcripts is strongly conserved between these distantly related species (Figure 2a). Conversely, 169 transcripts did not have significant matches in one or two of the species despite their strong similarity between chætognath and the remaining species. This lack of homologs is generally imputed to extensive gene loss [21]. Therefore, further comparisons were performed to identify genes whose homology assignment and gene loss in a peculiar lineage were unambiguous. Interestingly, the number of transcripts that did not match to one or more databases decreased from 169 to 74 when the complete set of sequences available for each bilaterian clade was employed as the database, instead of only one representative species (Figure 2b). The lack of homologous matches in some species could then be explained by an increase in evolutionary rates, which could have weakened the sequence similarity signal [29]. Additionally, the similarity level of matches increased when composite databases were employed (Figure 2), which supports the interest in this approach for phylogenomic reconstruction [18].

Two classes of genes provide reliable information for phylogeny inference (Figure 2b). Those that are highly shared between distantly related taxa constitute a set of conserved genes that are valuable markers for constructing phylogenomic datasets. In parallel, the genes that lack a homologous copy in one of the considered clades represent meaningful signature genes whose loss is attributable to a discrete event [23].

The candidates for signature genes are the genes inferred to be lost in one of the investigated clades (Figure 2b). Those candidates were carefully examined and their presence checked in the largest sets of available ESTs and full genome sequences. These data include the newly sequenced full genomes of lophotrochozoans and is assumed to include an exhaustive gene set in these species. Numerous candidate genes were invalidated because their homology relationships are disputable or because a homolog was retrieved from the full genome sequences surveyed. Among these candidates, the guanidinoacetate N-methyltransferase (GAMT) enzyme was recovered in the chætognath *S. cephaloptera*, in all studied deuterostomes, cnidarians and sister groups of metazoans (Figure S3 in Additional data file 1) but was not retrieved in any of the protostomes surveyed. Notably, this GAMT enzyme was also recovered in the acoel *Convoluta pulchra*, which was recently excluded from the protostomes [30]. This enzyme catalyzes the key step of creatine synthesis, an activity that was previously checked biochemically in a variety of organisms but was not found in selected protostomes [31]. GAMT was later noticed as missing in *D. melanogaster*, *Anopheles gambiae *and *Caeorhabditis elegans *genomes [32]. The presence of this ancient gene provides strong evidence for an early divergence of chætognaths from other protostomes. Indeed, the most parsimonious scenario states that this gene was lost in the protostome lineage after its split with chætognaths [18].

### Selection of marker genes for metazoan phylogeny

We attempted to evaluate the phylogenetic properties of the conserved genes that share equal levels of similarity with the main animal clades with respect to the convenience of their orthology assignment, their abundance in EST data and their molecular evolution properties. The main concerns when constructing phylogenomic-class datasets, especially from EST data, are the discarding of paralogous sequences, the removal of contaminants and the limitation of missing data. According to these criteria, we argue here that the set of RP genes is one of the best for setting up phylogenomic analysis in a large sample of taxa.

**Figure 3:**
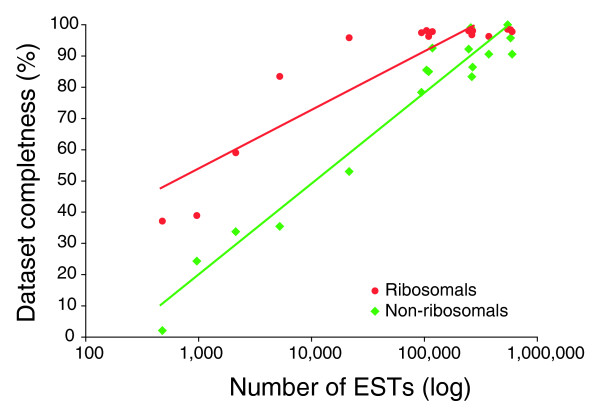
RP minimization of missing data in EST-based phylogenomic datasets. Dataset completeness was estimated for datasets composed of 78 RPs (red) or 115 other genes (green) retrieved from EST collections of a large range of sizes.

Among the 694 chætognath genes similar to a database entry, only 267 genes have homologs in the three main clades of bilaterians (score >150, Figure 2b). Copies of each selected marker were retrieved for all phyla studied for which EST data are available (Figure 3). In this way, the missing data were estimated through the occurrence of each gene in EST collections and preliminary phylogenetic analyses were carried out for all these independent alignments. Such controls unexpectedly highlighted putative paralogy problems for many candidate markers. If the orthologous transcript of a surveyed gene is missing in a non-exhaustive EST collection, a paralogous relative of this gene could be retrieved instead, with little chance of detection. Among candidate marker genes, RPs exhibit no ancient duplicates or out-paralogs and constitute a class of markers free from potential paralogy assignment problems [25,33]. Moreover, the gene-specific trees allowed detection of some contaminants in the EST collections, through the verification of unexpected clusterings in the tree (for example, several EST collections of parasitic organisms being contaminated by transcripts from their hosts).

Next, the amount of missing data was estimated using these raw alignments and compared with the number of ESTs in each available collection (Figure 3). The positive correlation observed between the number of ESTs and the completeness of the dataset is stronger when dealing with a dataset composed of RPs. For instance, the 5,235 EST collection of tardigrades yielded a dataset that is 77% complete for RPs, but only 35% complete for non-ribosomal markers. Thus, their large representation in EST collections strengthens the usefulness of RPs as phylogenetic markers.

### Chætognaths within renewed metazoan phylogeny

In order to assess the branching of chætognaths and to stress the usefulness of RP genes for phylogenomics, a RP dataset was assembled using the composite dataset approach [18]. This method depends on the selection of the least diverging copy of each marker gene in each taxon, such as a phylum, and thus allows reduction of the branch lengths of composite taxa (Table S1 in Additional data file 2). To overcome previous problems, both taxon sampling and inference methods were improved. Several new phyla were included in this analysis and, in particular, numerous protostome groups: priapulids, platyhelminthes, nermerteans, ectoprocts, entoprocts and rotifers [34-36]. Most rotifer sequences were retrieved from *Oryza sativa *(rice) ESTs, where they exist as contaminants, using their very specific splice-leader sequence as an anchor (see below and [37]). Rotifers constitute a key phylum with respect to chætognaths because they were sometimes grouped together in the gnathifera clade on the basis of morphological criteria [38]. Alternatively, a splitting of lophotrochozoans into two main lineages, the platyzoans (uniting platyhelminthes and rotifers) and the trochozoans (mainly annelids, molluscs, lophophorates and nermertes) has been proposed [39,40]. Otherwise, in addition to the traditional site-homogenous WAG model, we have assessed the phylogeny of bilaterians using the site-heterogeneous CAT model, which recently improved the limitation of the long-branch attraction artifact, a common pitfall in phylogenetic reconstruction [41,42]. The inclusion of the most recently released EST data for this large set of phyla led to a dataset including 11,730 amino acid positions and 25 taxa (Additional data file 4).

**Figure 4:**
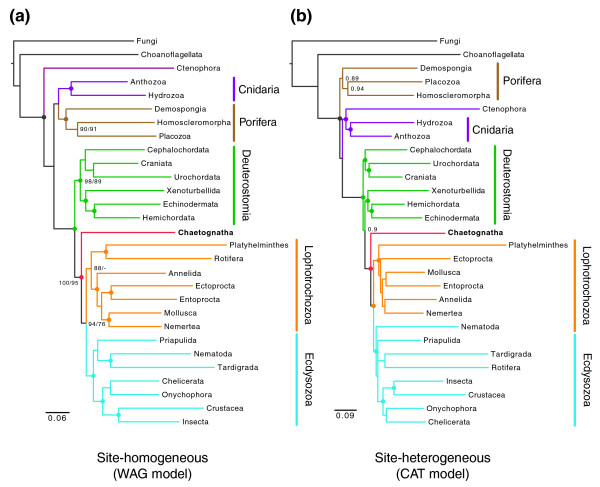
The basal-protostome branching of chætognaths is confirmed through improved inference methods and expanded taxon sampling. A RP alignment of 11,730 positions (after GBlock filtration; see Additional data file 4) was analyzed using two classes of models. **(a) **Site-homogeneous model (WAG) implemented in a maximum-likelihood framework (PhyML [80] and Treefinder [81]). Similar topology and maximal posterior probabilities were obtained with Bayesian analyses using the same model (MrBayes). **(b) **Site-heterogeneous model (CAT) implemented in a bayesian framework (Phylobayes [79]). Plain colored circles denote nodes for which significant support values were obtained (likelihood ratio statistics based on expected-likelihood weights (LR-ELW) >0.95 for site-homogenous and PP >0.95 for site-heterogenous). Support values are indicated for selected nodes: LR-ELW statistics and bootstrap (bold type) for maximum likelihood (ML) using the WAG model and posterior probabilities for Bayesian inference using the CAT model.

The analysis of this dataset confirmed the branching of chætognaths at the base of the protostomes with significant support values for both the site-homogeneous WAG model and the site-heterogeneous CAT model (bootstrap proportion (BP) of 76 and posterior probability (PP) of 1; Figure 4a,b). The inclusion of chætognaths within protostomes is still firmly supported (BP 95, PP 1; Figure 4). The inclusion of new taxa strengthens support for both the ecdyozoa and lophotrochozoa clades but the exact relationships within these two clades remain elusive [35,36,43]. Chætognaths and rotifers do not exhibit any peculiar affinities, prompting us to reject the gnathifera hypothesis [38]. Conversely, the branching of rotifers is problematic since this phylum is alternatively included in ecdysozoans and lophotrochozoans depending on the use of, respectively, site heterogeneous or homogeneous models (Figure 4). Thus, the clustering of platyhelminthes and rotifers in a platyzoa clade is supported by the WAG model but rejected by the CAT model, suggesting that this grouping may be somehow related to long-branch attraction (Figure 4). Alternatively, previous studies based on morphology and SSU genes have not argued for the ecdysozoan affinities of rotifers [38,39]. Surprisingly, CAT model analysis no longer succeeds in recovering the monophyly of the deuterostomes (Figure 4b). Instead, it provides limited support for the successive divergence of chordates and ambulacrarians (echinoderms and hemichordates; PP 0.9; Figure 4b). This striking topology was recovered by an independent study using the same heterogeneous CAT model [43] but was neither confirmed by WAG analyses (BP 89 for the monophyly of deuterostomes; Figure 4a) nor supported on morphological bases [34,38]. One can consider that the two unexpected branchings of rotifers and deuterostomes may be related to some artifact affecting the CAT model, such as sensitivity toward compositional biases [44]. Finally, the placozoan *Trichoplax adherens *surprisingly clustered within the poriferans, as a sister group of the homoscleromorphs (BP 91, PP 0.94; Figure 4), although this poriferan status has never been suggested before [45,46]. These challenging hypotheses will be investigated in further studies because they have deep implications for the evolution of metazoans (F Marlétaz *et al*., in progress).

Through extended taxon sampling and improved substitution models, these analyses strongly confirm our previous statements about basal-protostome branching of chætognaths [18] and exclude the basal-lophotrochozan hypothesis [19]. Although some areas of bilaterian trees are sometimes incongruent depending on models and inference methods, the position of chætognaths remains remarkably stable throughout our analyses. Furthermore, this branching is not only supported by the presence of GAMT, an unambiguous molecular signature, but also by the posterior Hox genes of chætognaths that are not related to the classes specific to ecdysozoans (Abd-B) or lophotrochozoans (Post1/2) [16]. Finally, this topology was also recovered by independent studies involving alternative gene and taxon sampling [30,35,43]. In a broader perspective, the strengthening of their phylogenetic position makes chætognaths a key model for comparative genomics among bilaterians.

### Genome duplication in the chætognath phylum

**Figure 5:**
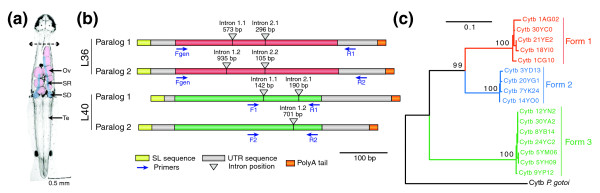
Alternative forms of selected markers amplified by PCR in order to assess the origin of polymorphism. **(a) **Localization of sperm within sperm receptacles (SR) and sperm ducts (SD) in the body of chætognath *S. cephaloptera *along with ovaries (Ov) and testis (Te). The double arrow indicates that head and body of individuals were split to perform independent PCR amplifications with the purpose of detecting possible contamination from the sperm genome. **(b) **Paralogous copies of nuclear genes RP L36 and L40 with their intron positions and average lengths, which are distinct in both cases (Additional data files 5 and 6). The names and positions of primers used for the amplification are also specified (Table S3 in Additional data file 2). **(c) **Relationships between alternative copies of Cytb retrieved within the ESTs with the three different forms detected by the designed primers (Additional data file 8). Boostrap proportions are indicated for selected nodes.

The clustering of similar sequences indicated that alternative nucleotide forms are present among the transcripts encoding the same protein. Two distinct forms are observed in most cases, although three forms encode some proteins. These forms are separated by a large amount of molecular divergence and can also be distinguished by their different 5' and 3' untranslated regions (UTRs), suggesting that they correspond to different genes (Figure 5 and Additional data files 5 and 6).

Ka/Ks ratios were calculated for all pairs of diverging forms to consider the impact of the nucleotide divergence on the protein sequences. The values of Ka/Ks range from 0.001-0.154 with a median value of 0.004, which confirmed the strong conservation of amino acid sequence despite the large synonymous substitutions observed in some cases (Ks values range from 0.8-75; Table S2 in Additional data file 2). These distinct forms were mainly retrieved for the most highly expressed genes, among which RP genes are prominent (Table 1). We verified that the observed molecular divergence could not be explained by the clustering of distant paralogous sequences. For the genes that have clear homologs among metazoans, the sequences of alternative forms always cluster together in phylogenetic analyses and are thus strongly separated from homologous genes of other animals. For instance, the RUX genes have undergone an ancient duplication resulting in the RUX-E and RUX-G paralogs in all metazoans. Interestingly, chætognaths display up to three forms of RUX-E and two forms of RUX-G, all these forms being closely related (Figure S4 in Additional data file 1; Additional data file 7).

**Table 1:** Occurrence of paralogous gene copies for ribosomal and non-ribosomal genes

	Ribosomal protein genes			Other genes		
		
Inferred duplicates	Gene number	Percent selected genes	Median EST number	Gene number	Percent selected genes	Median EST number
1	17	37%	12	14	70%	16
2	28	61%	23	3	15%	6
3	1	2%	23	3	15%	8.5
Total	46	-	20.5	20	-	8.5

Such a pattern could be explained by either the duplication of a large set of genes in the genome of chætognaths or, alternatively, it could be explained by the presence of cryptic species within the sampled population. In the first case, the observed differences would be attributed to the divergence between paralogous genes originating through the duplication, where the genome of one individual is expected to contain the two alternative nucleotide forms. In the second case, the observed genetic differences would be caused by the genetic divergence between the orthologous genes of several cryptic species spread among the population, where one individual is thus expected to contain only one of the alternative forms.

This cryptic speciation hypothesis may be supported by the strong polymorphism also observed for all genes of the mitochondrial genome, which constitutes an independent lineage from the nuclear genome. For example, cytochrome b (Cytb) transcripts but also cytochrome oxydase I and III are split into distinct forms separated by large molecular distances (Figure 5c; Figure S4 in Additional data file 1; Additional data files 8-10), thus testifying to the presence of distinct mitochondrial lineages within the sampled population.

To decide between these hypotheses, we designed a PCR screen to survey the alternative forms of selected markers in independent individuals. The genes for RPs L36 and L40 were targeted because they are nuclear genes displaying two alternative forms with the highest number of transcripts in the library (Table S2 in Additional data file 2). The mitochondrial Cytb gene served as an independent reference for the interpretation of results from nuclear genes. The three distinct forms of this strongly diverging mitochondrial gene were surveyed in all the individuals tested (Figure 5c). Chætognaths are hermaphroditic and, after fertilization, they store exogenous sperm in their sperm receptacles (Figure 5a), which makes it possible to amplify the DNA from another individual. Hence, in order to detect such contamination, we performed independent amplifications on heads, which are considered free from sperm contamination, as well as on the rest of the body, which contained sperm receptacles (Figure 5a). The experimental design made it possible to detect alternative forms through the amplification of specific DNA fragments of distinct sizes (Figure 5b; Table S3 in Additional data file 2). The PCR products were characterized by sequencing and nucleotide polymorphism was subsequently carefully examined. In addition to their nucleotide divergence in coding sequences, the distinct forms of nuclear genes for RPs L36 and L40 have alternative intron positions and lengths as well as differences in their 5' and 3' UTR regions (Figure 5b).

Performed on nine individuals, the amplifications revealed the presence of the two forms of the nuclear genes for RPs L36 and L40 in each individual (Table 2). Conversely, only one form of the mitochondrial Cytb gene was amplified in each individual with the exception of the body of individual 1, which includes two forms, thus suggesting contamination by exogenous sperm (Table 2). The amplification of the divergent nucleotide forms within one individual indicates that the alternative nucleotide forms correspond to paralogous nuclear copies originating through past gene duplication events (Table 1). Conversely, the alternative forms of the mitochondrial gene correspond to variation within the population. Because some genes, such as that encoding Translationally controlled tumor protein (TCTP), do not present paralogous copies despite their high expression levels (112 TCTP transcripts in the EST collection; Table S2 in Additional data file 2), we addressed the extent of these duplications in evaluating the quantity of duplicated genes. If the clusters of transcripts encoding the same protein include all the transcripts from alternative paralogous genes and if those paralogous genes have similar levels of expression, the probability that transcripts from these paralogous genes are represented in a given cluster is related to the size of this cluster (see Materials and methods). Hence, all the clusters that include more than six transcripts have at least a 95% chance of including transcripts from the two copies if they exist. Such clusters of transcripts were all checked for paralogous copies through sequence alignments and trees. Paralogs were detected within 35 of the 66 clusters investigated, which suggests that up to 69% of chætognath genes are the products of duplications. These paralogs could have arisen through either a whole genome duplication (WGD) event followed by an extensive gene loss, or several segmental duplication events.

**Table 2:** Distinct forms recovered from PCR amplification performed on heads and bodies of ten individuals for alternative marker genes

	L36		L40		Cytb		
			
Individual	1	2	1	2	1	2	3
1	+/+	+/+	+/+	+/+	/+	+/+	
2	+/+	+/+	+/+	+/+	+/+		
3	+/+	+/+	+/+	+/2	+		
4	+/+	+/+	2/2	+/2	+/+		
5	+/+	+/+	+/+	+/+			+/+
6	+/+	+/+	+/+	+/2	+/+		
7	+/+	+/+	+/+	+/+		+/+	
8	+/+	+/+	+/+	+/+	+/+		
9	+/+	+/+	+/+	+/+	+/+		

A plus sign indicates that one copy was amplified and a numeral indicates the number of copies if more than one were amplified (size distinct alleles). The copies amplified in heads and bodies are separated by a slash (head/body).

The hypothesis of a WGD event is reinforced by the high occurrence of RPs among duplicated genes (Table 1). The trend to retain RP genes was previously observed after WGD for *Paramecium tetraurelia*, yeast and plants [47-49] but is not a common occurrence in small-scale duplications. Conversely, it is difficult to understand why the paralogous genes have been retained after their duplication and maintained under purifying selection as emphasized by Ka/Ks values. This conclusion is in contradiction with the current view of gene destiny after genome duplication, which alternatively predicts that one of the gene duplicates is lost or undergoes the accumulation of substitutions [50]. Using a genome-level dataset, similar findings were made about the strongly duplicated genome of *Paramecium *where the retention of duplicated genes was accounted in part by dosage compensation constraints [47].

The most plausible dating is that this duplication occurred before the diversification of the major chætognath lineages. Two copies of SSU and LSU were retrieved in members of the phylum dispersed all over the tree of chætognaths [10-12]. Moreover, the survey of 226 ESTs available for *Flaccisagitta enflata *also revealed the presence of alternative nucleotide forms for some genes (data not shown), which would confirm that the duplication is not limited to SSU/LSU genes at this taxonomic scale. Further genome data would be required to date the duplication, for instance, in considering the Ks distribution of the set of paralogs [51], and also to definitively state the nature of the duplication through the analysis of synteny in duplicated blocks of the genome. Nevertheless, this preliminary transcriptomic survey stresses the usefulness of the chætognaths to study phylum-level genome duplication events and the destiny of paralogous genes.

### Population genomics

Beyond the molecular divergence between the coding sequences of duplicated paralogous genes, a subsequent survey of the genomic sequences of selected genes revealed that the level of polymorphism is strong within each paralogous gene (Table S4 in Additional data file 2). Multiple nucleotide substitutions as well as insertion/deletion events (indels) occurred within the introns of the four selected nuclear genes (paralogous copies of the genes for both RPs L36 and L40; Additional data files 11-14). Similarly, a large number of substitutions have accumulated in the various mitochondrial genes, thus revealing distinct mitochondrial lineages within the sampled population (Figure 5c; Figure S4 in Additional data file 1). However, these strong levels of divergence remain consistent with a population genetic structure because of the regular AT composition and the limited degree of saturation revealed by Ts/Tv ratios, singleton positions being essentially transition substitutions (Table S4 in Additional data file 2; Figure S6 in Additional data file 1).

**Figure 6:**
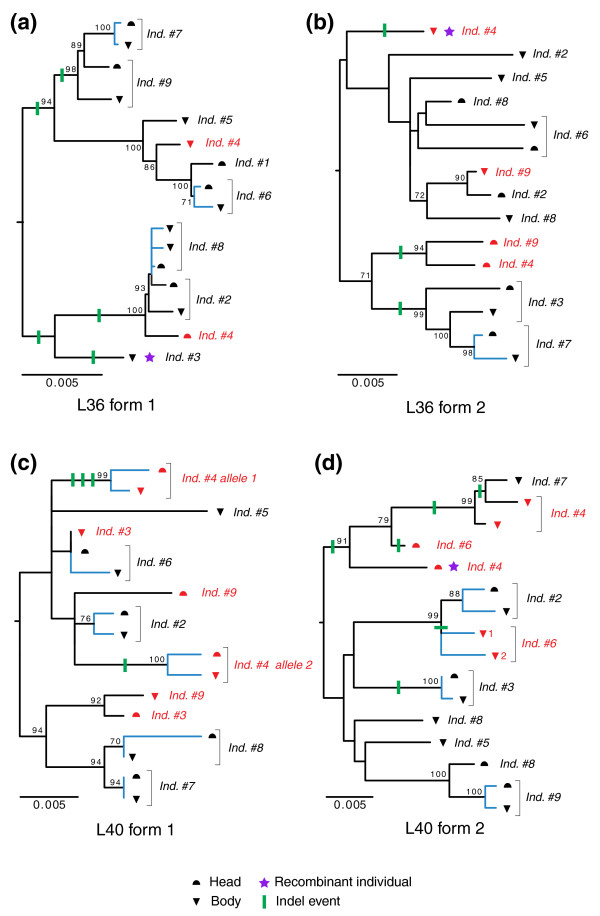
Relationships between haplotypes of nine individuals, including distinct head and body sequences for four marker genes, including two pairs of paralogous sequences: **(a) **L36 form 1; **(b) **L36 form 2; **(c) **L40 form 1; **(d) **L40 form 2. The indels are plotted onto the branches (green lines). Noticeably, some individuals display mixed sequences from different haplotypes, which is explained by recombination events between alleles (purple star). The substitutions occurring between copies of the same allele in the head and body of individuals (blue branches) are assumed to be somatic mutations. These neighbor-joining trees were inferred assuming kimura 2 parameter distances from Additional data files 7-10. Boostrap proportions are indicated for selected nodes.

We attempted to determine the origin of this population genetic heterogeneity, which could, for instance, be due to a cryptic speciation or to a past hybridization. For this, the sequences of each individual were compared using phylogenetic trees and indels as discrete informative characteristics (Figure 6). For each marker gene, individual sequences split into several major clades supported by strong bootstrap and discrete indel events, which allows unambiguous identification of heterozygous individuals (Figure 6). For example, individual 4 is heterozygous for all markers and individuals 6, 9 and 3 are heterozygous for at least one marker. Moreover, the occurrence of several cases of putative recombinations between alleles highlights the heterozygous status of some individuals (individuals 3 and 4, Figure 6b,d). Notably, our PCR-based experimental design provided positive evidence only for heterozygosis because two amplifications (head and body) were carried out per individual, yielding 0.5 probability to detect heterozygosity. Heterozygous individuals could thus be even more abundant than observed. These heterozygous cases convincingly demonstrate that a shuffling occurs between the most divergent alleles of each gene, which constitutes strong evidence for interbreeding within the sampled population. This finding definitely excludes the possibility of cryptic speciation within this *S. cephaloptera *population. Alternatively, the panmixy hypothesis was confirmed by the unimodal distribution of pairwise divergences in mismatch analysis, which is consistent with constant population size and excludes a past hybridization event (Figure S6 in Additional data file 1). Finally, the distinct mitochondrial lineages are spread within the population but they are not correlated with any haplotype differentiation at the nuclear level, which is a strong argument against the cryptic speciation hypothesis. This type of mitochondrial diversity was previously discovered for the planktonic species *Sagitta setosa *but was also interpreted with difficulty [52].

Strikingly, these comparisons also highlighted molecular divergence between the head and the body of some individuals for each of the five markers investigated (Figure 6 and Additional data file 4). Such substitutions cannot be explained by a heterozygous status of those individuals because sequences from head and body were firmly clustered in the tree (Figure 6). For example, individual 4 exhibits well-separated alleles present in both head and body but intra-individual substitution took place between head and body for both of these alleles (Figure 6c). This pattern of substitutions may be explained by the occurrence of somatic mutations during the life of individuals. This interpretation is corroborated by the large extent of intra-individual substitutions in all marker genes and all individuals. Somatic mutations are considered as rare conditions, mainly known from related disorders in humans [53]. Less clear are the evolutionary implications and putative benefits of this phenomenon [54]. They are sometimes suspected to play a prominent role in apoptosis and possibly in the regulation of cell division [54]. Moreover, somatic mutations have been demonstrated to be more widespread in *Drosophila *than in mammals [55], and are sometimes correlated with extensive chromosome rearrangement in the *Drosophila *lineage [56]. However, little is known about the extent and importance of this process in the non-model organisms. In the case of the chætognath, somatic mutation could be due to the high mutation rates that seem to affect both germline and soma and could explain the divergence at the population and individual levels. The possible relationship of these accelerated mutation rates with structural reshaping of the genome after duplication deserves further evaluation.

Notably, this level of somatic mutation generates a strong background noise that hinders the accurate interpretation of point mutations related to the diversity of haplotypes. Moreover, traditional hypotheses of population genetics are challenged by our findings: the genetic distances observed between individuals of a single population reach species-level without any evidence for cryptic speciation or past hybridization. In parallel, multiple mitochondrial lineages diverge and are spread and maintained within a single population [52]. If such features are revealed as more widespread than expected, the data collected from numerous population genetic or phylogeographic studies require more cautious interpretation. This observation pleads for an increase of the sampling depth for population genetics, especially through genomic approaches [57].

### Trans-splicing transcript maturation in chætognaths

**Figure 7:**
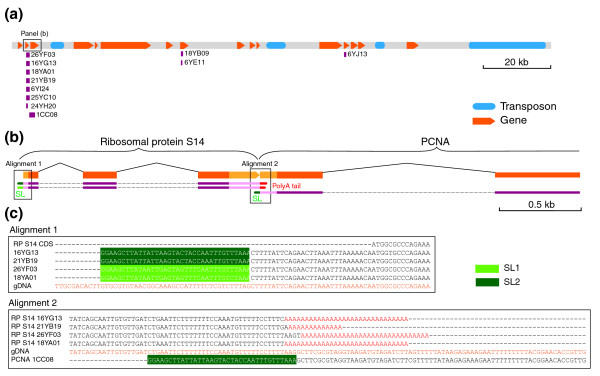
Identified *S. cephaloptera *operon within the BAC 35YA21. **(a) **Structure of the 158 kb BAC 35A21, including the predicted genes and the mapping of ESTs that bear SLs (purple). Detailed EST/BAC alignments are provided as Additional data files 15-17. **(b) **Detailed structure of the identified chætognath operon with RP S14 and PCNA genes and the corresponding ESTs (purple) that exhibit SL sequences. UTR (genomic, light orange; EST, light purple) and coding sequences (genomic, orange; EST, purple). **(c) **Alignments of selected regions of the operon, beginning and end of genes showing genomic DNA (orange) and transcripts with their alternative SL forms. The very short distance encountered between the end and beginning of the two genes argues for polycistronic transcription.

The survey of the chætognath EST collection detected a common 36 nucleotide motif shared at their 5' end between transcripts from unrelated genes. Selected cases showed that these short motifs are absent from upstream genomic regions of the genes (Figure 7c; Additional data files 15-17). These findings strongly suggest that mRNA of *S. cephaloptera *undergoes trans-splicing maturation. This mRNA processing occurs through the spliceosomal transfer of a small RNA molecule at the 5' end of the mRNA from an independent spliced-leader (SL) gene and has been described in numerous species [26]. To exclude the possibility of an artifact, the 226 available EST sequences of *F. enflata*, another chætognath species, were screened using the identified SL sequences from *S. cephaloptera*. This survey led to the recovery of similar SL sequences at the 5' end of several cDNA sequences. As *F. enflata *and *S. cephaloptera *belong to the two main orders of chætognaths [10], the trans-splicing mechanism is likely to be present in the whole chætognath phylum.

An extensive study of trans-splicing in the nematode phylum has previously revealed the presence of different forms of SL sequences [58]. Similarly, several different SL sequences were retrieved from *S. cephalotera *(Table 3). This number is consistent with the strong level of polymorphism previously observed for coding sequences but two distinct SL forms alone (SL1 and SL2) represent 87% of the SL sequences (Table 3). These forms do not exhibit any specificity for the different paralogs since SLs are added randomly to transcripts from these paralogs (Figure 7; Additional data files 5 and 6). In an attempt to understand the evolutionary history of trans-splicing within the chætognath phylum, the SL forms of *F. enflata *were compared with those of *S. cephaloptera*. This set of chætognath SL sequences splits into four main forms: two of them, SL1 and SL3, are present in both the species investigated whereas the two other forms, SL2 and SL4, are specific for *S. cephaloptera *and *F. enflata*, respectively (Table 3). Otherwise, neither of these chætognath SL forms display similarity with the SL of another phyla. This finding suggests that just as in nematodes, the evolution of alternative forms could have occurred at a relatively reduced taxonomic scale [58].

**Table 3:** Splice-leader isoforms isolated in two chætognath species: *S. cephaloptera *and *F. enflata*

Form	ID	Sequence	Species	Abundance
**Form 1**	**SL1**	GGAAGCTAAAATTCTTTTA--TTTGCTT-AATTAAA	Both	<
				
**Form 2**	**SL2.0**	GGAAGCTTATAATTGAGTAGTTTCAATTTGTTTAAA	Both	70.83%
	SL2.1	GGAAGCTTATAATTGAGTGGTTTCAATTTGTTTAAA	*S. cephaloptera*	<
	SL2.2	GGAAGCTTATAATTGAGCAGTTTCAATTTGTTTAAA	*S. cephaloptera*	<
	SL2.3	GGAAGCTTATAATTGACTATTTTCAATTTGTTTAAA	*S. cephaloptera*	<
	SL2.4	GGAAGCTTATAATTGAGTATTTTCAATTTGTTTAAA	*S. cephaloptera*	1.44%
	SL2.5	GGAAGCTTATAATTGCGTATTTTCAATTTGTTTAAA	*S. cephaloptera*	<
	SL2.6	GGAAGCTTATAATTGCGTAGTTTCAATTTGTTTAAA	*S. cephaloptera*	<
	SL2.7	GGAAGCTTATAATTCATTAGTTTCAATTTGTTTAAA	*S. cephaloptera*	<
	SL2.8	GGAAGCTTATAATTCAGTAGTTTCAATTTGTTTAAA	*S. cephaloptera*	<
	SL2.9	GGAAGCTTATAATTGATTAGTTTCAATTTGTTTAAA	*S. cephaloptera*	2.17%
	SL2.10	GGAAGCTTATAACTGTTTAGTTTCAATTTGTTTAAA	*S. cephaloptera*	<
	SL2.11	GGAAGCTTATAATTGACTAGTTTCAATTTGTTTAAA	*S. cephaloptera*	<
	SL2.12	GGAAGCTTATAATTGAGTAGCTTCAATTTGTTTAAA	*S. cephaloptera*	<
				
**Form 3**	SL3.1	GGAAGCCAAT-TTCTACTA-CTTCACTT-GTTTAAA	*F. enflata*	-
	SL3.2	GGAAGCTAAT-TTCTACTA-CTTCACTT-GTTTAAA	*F. enflata*	-
	SL3.3	GGAAGCTAAT-ATCTACTA-CTTCACTTTGTTTAAA	*F. enflata*	-
				
**Form 4**	SL4.1	GGAAGCTTATTATCAAGTACTACCAATTTGTTTAAA	*S. cephaloptera*	<
	SL4.2	GGAAGCTTATTATTAAGTACTACCAATTTGTTTAAA	*S. cephaloptera*	16.91%
	SL4.3	GGAAGCTTATTATTAAGTACTACCAAATTGTTTAAA	*S. cephaloptera*	<
	SL4.4	GGAAGCTTATTATTAAGTACTACCAGTTTGTTTAAA	*S. cephaloptera*	<
	SL4.5	GGAAGCTTATTATTACGTACTACCAATTTGTTTAAA	*S. cephaloptera*	<
	SL4.6	GGAAGCTTATTATTAATTACTACCAATTTGTTTAAA	*S. cephaloptera*	<
	SL4.7	GGAAGCTTATTATTATTTACTACCAATTTGTTTAAA	*S. cephaloptera*	<
	SL4.8	GGAAGCTTATTATTAACTACTACCAATTTGTTTAAA	*S. cephaloptera*	<
	SL4.9	GGAAGCTTATTATTCAGTACTACCAATTTGTTTAAA	*S. cephaloptera*	<

The abundance of the forms corresponds to the rate of the forms among all SLs and is calculated only for *S. cephaloptera *sequences. A 'less-than' sign indicates that the abundance is below 1%.

**Figure 8:**
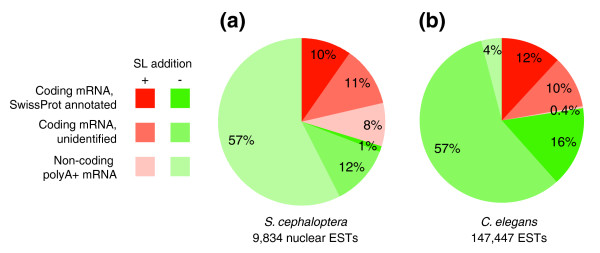
Categories of trans-spliced transcripts for chætognath **(a) ***S. cephaloptera *and **(b) **nematode *C. elegans*. The presence of a SL sequence is related to coding properties and homologous matches in SwissProt (score >150) of the sequences. *C. elegans *exhibits less non-coding transcripts than *S. cephaloptera*.

Within the EST collection, 2,914 sequences exhibit SL addition, which represents 30% of nuclear transcripts (Figure 8a). Among the SL population, 72% are coding transcripts, of which 46% have a homolog in SwissProt. The clustering of similar coding trans-spliced transcripts indicated that 41% of putative genes undergo SL addition. Furthermore, the relationship between trans-splicing and expression level was tested through the comparison of the number of ESTs per cluster of trans-spliced or non-trans-spliced transcripts. If we posit that this number can be considered as an estimate of the expression level, trans-spliced genes are significantly more expressed than others (Wilcoxon rank test, *p *< 2.2e^-16^). For instance, among the 50 more expressed genes (that is, biggest EST clusters), only two are not trans-spliced. These values suggest that trans-splicing is involved in the regulation of a set of strongly expressed genes responsible for key cellular functions, for example, the RP set.

Hence, the set of trans-spliced genes of *S. cephaloptera *was compared with those of other animals that carry out this RNA maturation process (Table 4). Particularly, trans-splicing was characterized in the nematode *C. elegans *model, for which a large EST set is available [58,59]. These data were especially useful for extensive comparisons with *S. cephaloptera *(Figure 8b). The amount of ESTs with a SL is slightly smaller in *C. elegans *(22%) than in *S. cephaloptera *(29%) and trans-spliced transcripts are missing from the very reduced non-coding ESTs, only 5% of the total EST collection in *C. elegans*. This comparison suggests that trans-splicing is at least as widespread in *S. cephaloptera *as in *C. elegans*. Furthermore, the set of genes affected by trans-splicing in *S. cephaloptera *was comparable with those of *C. elegans*. Among the 119 different genes that are both trans-spliced and annotated using SwissProt with high confidence, 79 have trans-spliced homologous genes in *C. elegans *(Additional data file 3). These values do not include the 78 RP set, which are conserved and trans-spliced in both species. While the molecular actors involved in trans-splicing do not exhibit evolutionary conservation [26], the genes undergoing this kind of processing could otherwise be similar in distantly related species.

**Table 4:** Distribution and modalities of trans-splicing among metazoans

Phylum: species	Trans-splicing	Percent trans-spliced genes	Number of SL forms	Operons	Percent genes in operons	References
Cnidaria: *Hydra vulgaris*	+	Very few*	2	?	NA	[85]
Urochordata (tunicate): *Ciona intestinalis*	+	~2%*	1	+	~2%	[61]
Chætognatha: *Spadella cephaloptera*; *Flaccisagitta enflata*	+	~55%	2*	+	ND	This study
Acoela: *Convoluta pulchra*	+	>5%	1	?	ND	This study
Rotifera: *Adineta ricciae*; *Philodina *sp.	+	ND	1	ND	ND	[37]
Platyhelminth: *Schistosoma mansoni*; seven more species^‡^	+	>3%^†^	1-2	+	ND	[60,62]
Nematoda: *Caenothabditis elegans*	+	~50%	2	+	~20%	[58,59]

*This figure was obtained by the analysis of a sample of several cDNA libraries sequenced from the 5' end. ^†^This figure was obtained from 3' end sequenced cDNA libraries and may be greatly underestimated. ^‡^For a precise list of these species see [62]. NA, not applicable; ND, not determined.

Trans-splicing has been discovered in a set of organisms spread all over the phylogenetic tree of eukaryotes, but the molecular actors involved in this RNA maturation process do not exhibit any evolutionary conservation: the SL sequences cannot be aligned and the ribonucleoproteic machineries involved in this process have no homologs in other species [26,37,60-62]. However, the discovery of SL trans-splicing in new animal phyla strongly argues for an ancient origin of this process, especially if these phyla have significant phylogenetic positions, such as chætognaths or acoels. Noticeably, we also found evidence for SL addition in recently released ESTs from the acoels (unpublished observations), worm-like animals that were recently excluded from the protostomes [30]. Moreover, an extensive comparison of available genomic data revealed that the set of genes undergoing trans-splicing is evolutionarily conserved between those species, which is another indication of a putative common evolutionary origin of trans-splicing (Additional data file 3 and Table 4).

### Operonic transcription in *S. cephaloptera*

In the nematode chætognath *C. elegans *and the tunicate *Ciona intestinalis*, polycistronic mRNA molecules that contain two or more genes are transcribed from operon structures and subsequently resolved by trans-splicing [58,59]. We mined for similar eukaryotic operons in our pool of sequenced bacterial artificial chromosomes (BACs) in looking for clusters of genes with the same transcription orientation and very short intergenic regions. The trans-splicing maturation of these putative operons was confirmed by mapping the tran-spliced ESTs onto these genomic sequences. One operon including RP S14 and PCNA genes was successfully identified in the BAC 35A21 that otherwise contains a large number of genes undergoing trans-splicing (Figure 7a; Table S5 in Additional data file 2; Additional data files 15-17). EST mapping and *ab initio *gene prediction suggested that only three bases separate the polyadenylation site of the upstream RP S14 gene from the acceptor splice site at the PCNA downstream gene. This distance is even shorter than that in *C. intestinalis *operons [61] and clearly excludes the possibility for a re-initiation of transcription between the genes (Figure 7c). This BAC sequence contains several other predicted genes that are closely clustered, share the same orientation and could thus belong to operonic structures (Figure 7; Table S5 in Additional data file 2). The transcripts of gene RP S14 include the two major SL 1 or 2 forms (Figure 7c). Similarly, transcripts from both paralogous copies of duplicated genes bear any of the alternative SL forms. Hence, contrary to *C. elegans *for which a SL form is specific for the upstream and the downstream genes in operons [58], no specificity of the SL for gene position in the operon was recovered in *S. cephaloptera *(Figure 7).

Hence, we have provided arguments for polycistronic transcription in another animal phylum, the chætognaths. Animal operons have previously been described in the nematodes and in the tunicate *C. intestinalis *and were often interpreted as an adaptation to the genome size reduction observed in both these organisms [26]. However, the genome size of *S. cephaloptera *is rather large at 1.05 Gb [63] and the transcriptome of chætognaths is conserved among bilaterians, in terms of gene similarity and content. Furthermore, extensive study of genes co-transcribed in *C. elegans *led to the conclusion that the operons constitute functional units in grouping genes involved in similar pathways [59]. Hence, the presence of operonic transcription in the chætognath, a basal protostome, strongly suggests that this mechanism should be more likely considered as an original mode of gene regulation, whose evolutionary importance may have been underestimated. Thanks to the pivotal comparative genomic abilities of chætognaths, the comparison of operon structure and content between nematodes and chætognaths should allow the extension of the functional results obtained in *C. elegans *to a larger set of organisms, including vertebrates. Such an approach would require further genomic data in the chætognath but will likely lead to the characterization of new gene regulatory networks, potentially leading to a better understanding of some genetic disorders [59].

## Conclusion

Chætognaths have been formerly known for their peculiar morphological characteristics. The fine-scale analysis of transcriptomic data reported here illuminates significant genomic features of this phylum, strengthening its very original status among bilaterians. These genome features may be shuffled into opposite categories: shared ancestral characteristics of bilaterians on one hand and lineage-specific rearrangements and divergences on the other. First, the chætognath transcriptome bears a core set of genes conserved within bilaterians. Together with recent evidence from lophotrochozoans such as annelids [64], this result confirms that the extensive gene loss previously observed within insects or nematodes cannot be extended to the whole protostome lineage. The conservation of such a core gene set was exploited for phylogenomic inference through the definition of RP genes as high-grade marker genes for phylogeny. Subsequent analyses of these marker genes confirmed the basal position of chætognaths among protostomes, a position that has been previously supported [18,30,43]. This position strongly impacts on the understanding of the evolution of embryological characteristics since it rejects some developmental characteristics at the stem of bilaterians, such as enterocoely or secondary mouth opening that were considered to be deuterostome-specific.

This interpretation can be extended to the new genome-level characteristics that we have highlighted here. For example, the trans-splicing mechanism of mRNA processing as well as the operonic transcription depicted here were often interpreted as a secondary evolved condition related to peculiar adaptations in animals such as nematodes or tunicates [26]. Contrarily, the occurrence of these mechanisms in chætognaths and acoels suggests that their evolutionary origins may be more ancient than originally expected. Together, these findings strongly argue for the relevance of chætognaths as a comparative genomics model because of their conserved gene set, their original phylogenetic position and their original features such as trans-splicing and operonic transcription. For example, the study of chætognath operons could be a promising approach to identify molecular pathways involved in human genetic diseases [59].

Some genetic and genomic features of chætognaths we have illustrated here are unique among the animals. We have discovered a genome duplication event followed by a high retention of duplicated genes that were maintained under strong purifying selection. The current view of the evolution of duplicated genes in animals or plants mainly predicts a loss of genes that have not evolved novel functions [50,65]. This view is contradicted by recent evidence from protists [47], plants [25] and the whole fungal kingdom [66] that suggests new modalities of genome evolution and, in particular, strong retention and constraint of former duplicated genes. Hence, the chætognath genome duplication deserves further documentation and investigation as one possible special case among bilaterians. Another unique feature of chætognaths is the high level of divergence within the population. We demonstrated that this divergence is related to neither cryptic speciation nor past hybridization but conversely revealed very high mutation rates within the germ line of individuals but also within their soma. These observations have deep implications for the correct interpretation of numerous population genetics studies. This apparent genome plasticity with duplications and high mutation rates is in contrast to the strong morphological conservation of the phylum that has continued nearly unchanged since the early Cambrian period [8,9]. This uncoupling between morphological conservation and genome plasticity makes us wonder how the gene regulatory networks responsible for the establishment of the body organization of these animals could have remained stable despite such a genome dynamic?

In summary, these findings promote the study of chætognath for orienting not only morphological but also genomic characteristics among bilaterians. With the recent rise of lophotrochozoans as promising model systems [67], the chætognath phylum represents the next step of investigation within protostomes with its striking combination of distinct ancestral and derived features that could illuminate the evolution of bilaterians.

## Materials and methods

### cDNA library and EST sequencing

The juvenile-staged cDNA library of *S. cephaloptera*, Bush 1851 was previously described in [18]. Briefly, polyA+ RNAs were isolated from hatchling to one-day-old juveniles collected in the coastal area near Marseille (Le Brusc, La Ciotat). After reverse transcription and selection of longer transcripts by size fractionation, cDNAs were cloned into Lambda Triplex 2 vector (Clontech, Palo Alto, CA, USA). There were 12,324 clones sequenced from a 5' primer at Génoscope (CNS, France). A total of 390 sequences were discarded due to vector contamination or cloning artifacts, yielding a total 11,934 sequences.

### BAC library

The BAC library was constructed by BioS&T Inc. (Montreal, Québec, Canada) in the pIndigoBAC-5 vector (Epicentre, Madison, WI, USA) from adult *S. cephalotera *genomic DNA. Average insert size is 135 kb. The library was arrayed and selected clones were sequenced using a shotgun approach at Génoscope.

### Analysis of the EST collection

The sequences were annotated through Blast searches [68] against SwissProt and TrEMBL databases. The transcripts were searched for reliable coding sequences using ESTScan and subsequently sorted into coding and non-coding sequences [69]. Transcripts from the same putative genes were grouped into clusters using CLOBB [70]. The functional Gene Ontology classification was retrieved from SwissProt matches using Fatigo [71]. Transcripts that bear splice-leader at their 5' extremity were searched with Blastn using the SL sequences obtained from a preliminary screen. Data parsing processes were conducted using Perl scripting language and the Bioperl resource [72].

### Databases and searches for marker genes

The set of databases compared with the chætognath transcriptome includes the transcriptomes of *D. melanogaster *and *H. sapiens *downloaded from Ensembl [73]. The partial transcriptome of *Lumbricus rubellus *as well as clade-level EST databases were obtained from the NCBI EST database using appropriate keywords [74]. The recently released genomes of several lophotrochozoans (*Capitella capitata, Helobdella robusta, Lottia gigantea, Aplysia californica*, *Schmidtea mediterranea *and *Schistosoma mansoni*) were accessed directly on the JGI web site [75] or retrieved from the NCBI trace archive [76]. The respective scores were visualized using Simitri [77].

### Duplicated gene detection

All the transcript sequences encoding similar protein coding sequences were clustered on the basis of a similar SwissProt hit or a reciprocal blast hit after conceptual translation by ESTScan [69]. Gene clusters were analyzed using Clustalw alignment, phylogenetic analyses and visual checking for the inclusion of potential diverging sequences (Table S1 in Additional data file 2). The minimal EST number (N) per cluster to detect the occurrence of two duplicated paralogous sequences at a 5% probability is 6 as estimated by 1 - 2(1/2)^N^. Ka/Ks were computed using PAML [78].

### Phylogenetic analyses

The RP dataset was built using a composite database approach so as to select the least divergent sequences for each taxon [18]. The species set involved in each taxon as well as the data on missing sequences are available in Table S1 in Additional data file 2. This dataset was analyzed using the CAT model as implemented in Phylobayes [79]. Maximum-likelihood and Bayesian inferences were performed using PhyML [80], Treefinder [81] and MrBayes [82], respectively, and both assumed a WAG+Γ4+I model. For bayesian analyses (MrBayes and Phylobayes), at least two chains were launched, their convergence was verified and the corresponding burn-in period discarded.

### PCR amplifications of duplicated markers

The individuals of *S. cephaloptera *employed for polymorphism analysis were collected across the *Posidonia *seagrass, in the calanque of Sormiou, in the coastal area near Marseille, France. Copies of nuclear RP S36 and RP L40 and mitochondrial Cytb genes were amplified by PCR using allele-specific primers (Table S3 in Additional data file 2). Genomic DNA extractions were carried out on heads and bodies of *S. cephaloptera *individuals from the calanque of Sormiou using the Wizard SV Kit (Promega, Madison, WI, USA) and PCR reactions were carried out using GoTaq polymerase (Promega). PCR conditions were defined according to the melting temperature determined for each pair of primers. Fragments were subsequently gel-purified, cloned in pGEM-T easy (Promega) and sequenced. PCR artifacts were excluded by two approaches: first, amplifications were performed on head and body of the same individual, which allows checking of the congruence between the sequences; then, the accuracy of DNA polymerase was verified by amplifying and sequencing cloned DNA. These validations were confirmed by the recovery of the amplified sequences in the EST library and by the high levels of substitutions between sequences, which are too large to be interpreted as PCR artifacts.

### Population genetic analyses

Pairwise sequence comparisons and molecular evolution analyses were performed with Mega2 software assuming the kimura 2 parameters model [83]. The demographic history of the populations was evaluated by mismatch analyses using Tajima's D and Fu and Li's D neutrality test implemented in DNAsp software [84].

### Accession numbers

The set of sequences analyzed in this paper has been deposited in GenBank with the following accession numbers: CU555081-CU563075 and CU693608-CU693592 for the 11,934 ESTs, CU672232 for the BAC 35YA21, and EU529890-EU529968 for the sequences of RP L36 and L40 paralogs in nine individuals (Table 2).

## Abbreviations

BAC, bacterial artificial chromosome; BP, bootstrap proportion; Cytb, cytochrome b; EST, expressed sequence tag; GAMT, guanidinoacetate N-methyltransferase; indels, insertion or deletion events; LSU, large subunit of ribosomal RNA; PP, posterior probability; RP, ribosomal protein; SL, splice-leader; SSU, small subunit of ribosomal RNA; UTR, untranslated region; WGD, whole genome duplication.

## Authors' contributions

YLP and FM conceived the project and performed the analyses focused on phylogenomics, characterization of duplication and trans-splicing. YLP and XC were in charge of the construction of the cDNA and BAC libraries. YLP, FM, and AG carried out the experiments related to the study of polymorphism in the population. YP ensured the collection of animals required for construction of libraries and population genetics studies. PW supervised the sequencing project that SS, CD and GG carried out. FM wrote the paper and all authors agreed on the final version.

## Additional data files

The following additional data are available. Additional data file 1 includes supplementary Figures S1-S6 with legends: EST library statistics (Figure S1), Gene Ontology annotation (Figure S2), alignment of GAMT in selected taxa (Figure S3), trees illustrating the divergence between copies of nuclear and mitochondrial genes in the library (Figure S4), transition versus transversion ratios for the five targeted genes (Figure S5), and mismatch analyses and testing for the five targeted genes (Figure S6). Additional data file 2 includes supplementary Tables S1-S5 with legends: composition of the composite taxa involved in phylogenomic analyses (Table S1), list of all clusters of transcripts corresponding to alternative forms or not with Ka/Ks ratios (Table S2), list of primers employed for PCR amplifications of alternative forms (Table S3), comparison of molecular evolution trends for four genes retrieved in nine individuals (Table S4), and annotation of the BAC 35A21 (Table S5). Additional data file 3 lists the trans-spliced genes conserved between *S. cephaloptera *and *C. elegans *andhomologous to a SwissProt entry (Figure S6 in Additional data file 1). Additional data file 4 is the concatenated gblocked alignment that clusters 77 RPs, yielding 24 taxa and 11,730 shuffled amino acid positions. This dataset was employed for phylogenomic reconstruction. Additional data files 5 and 6 are the transcript sequences of RP L36 (Additional data file 5) and RP L40 (Additional data file 6) exhibiting the alternative forms corresponding to paralogous copies and the position of primers designed to specifically amplify these forms. Additional data file 7 is the alignment of amino acid sequences of RUX paralogs and variants from *S. cephaloptera *and some other eukaryotic taxa. Additional data file 8 is the alignment of Cytb displaying selected variants retrieved from the EST library and the coding sequences of Cytb from nine individuals showing the differences between genetic lineages within the population (primers employed for the specific amplifications are included). Additional data files 9 and 10 are the alignments of cytochrome oxydase I and III transcripts retrieved from the EST library. Additional data files 11, 12, 13 and 14 are the alignments of genomic sequence of paralogs of RP L36 (paralogs 1 and 2, Additional data files 11 and 12) and RP L40 (paralogs 1 and 2, Additional data files 13 and 14) from 9 individuals that have been amplified from the body (B) or head (H) of individuals as indicated in the sequence name. Additional data files 15, 16 and 17 are EST/BAC alignments for four trans-spliced genes present in BAC 35A21, whose transcripts are represented in the EST library. Additional data file 15 contains the RP S14 and PCNA genes gathered in an operonic structure and eight matching ESTs. Additional data file 16 contains the COP9 gene and two matching ESTs. Additional data file 17 contains an unidentified gene matching EST clones 6YJ23. All these alignments clearly exhibit lack of a SL motif in the genomic sequence.

## Supplementary Material

Additional data file 1 Figure S1: EST library statistics. Figure S2: Gene Ontology annotation. Figure S3: alignment of GAMT in selected taxa. Figure S4: trees illustrating the divergence between copies of nuclear and mitochondrial genes in the library. Figure S5: transition versus transversion ratios for the five targeted genes. Figure S6: mismatch analyses and testing for the five targeted genes. Click here for file

Additional data file 2 Table S1: composition of the composite taxa involved in phylogenomic analyses. Table S2: list of all clusters of transcripts corresponding to alternative forms or not with Ka/Ks ratios. Table S3: list of primers employed for PCR amplifications of alternative forms. Table S4: comparison of molecular evolution trends for four genes retrieved in nine individuals. Table S5: annotation of the BAC 35A21. Click here for file

Additional data file 3 Trans-spliced genes conserved between *S. cephaloptera *and *C. elegans *and homologous to a SwissProt entry (Figure S6 in Additional data file 1). Click here for file

Additional data file 4 This dataset was employed for phylogenomic reconstruction. Click here for file

Additional data file 5 Transcript sequences of RP L36 exhibiting the alternative forms corresponding to paralogous copies and the position of primers designed to specifically amplify these forms. Click here for file

Additional data file 6 Transcript sequences of RP L40 exhibiting the alternative forms corresponding to paralogous copies and the position of primers designed to specifically amplify these forms. Click here for file

Additional data file 7 Alignment of amino acid sequences of RUX paralogs and variants from *S. cephaloptera *and some other eukaryotic taxa. Click here for file

Additional data file 8 Primers employed for the specific amplifications are included. Click here for file

Additional data file 9 Alignment of cytochrome oxydase I transcripts retrieved from the EST library. Click here for file

Additional data file 10 Alignment of cytochrome oxydase III transcripts retrieved from the EST library. Click here for file

Additional data file 11 Sequences were amplified from the body (B) or head (H) of individuals as indicated in the sequence name. Click here for file

Additional data file 12 Sequences were amplified from the body (B) or head (H) of individuals as indicated in the sequence name. Click here for file

Additional data file 13 Sequences were amplified from the body (B) or head (H) of individuals as indicated in the sequence name. Click here for file

Additional data file 14 Sequences were amplified from the body (B) or head (H) of individuals as indicated in the sequence name. Click here for file

Additional data file 15 All the alignments in Additional data files 15, 16, 17 clearly exhibit lack of a SL motif in the genomic sequence. Click here for file

Additional data file 16 All the alignments in Additional data files 15, 16, 17 clearly exhibit lack of a SL motif in the genomic sequence. Click here for file

Additional data file 17 All the alignments in Additional data files 15, 16, 17 clearly exhibit lack of a SL motif in the genomic sequence. Click here for file
