# Supplementary material for: Chætognath transcriptome reveals ancestral and unique features among bilaterians
Source: Genome Biol. 2008 Jun 4;9(6):R94. doi: 10.1186/gb-2008-9-6-r94 (PMC2481426; doi:10.1186/gb-2008-9-6-r94)
Supplement: Additional data file 1 — Figure S1: EST library statistics. Figure S2: Gene Ontology annotation. Figure S3: alignment of GAMT in selected taxa. Figure S4: trees illustrating the divergence between copies of nuclear and mitochondrial genes in the library. Figure S5: transition versus transversion ratios for the five targeted genes. Figure S6: mismatch analyses and testing for the five targeted genes. [file gb-2008-9-6-r94-S1.pdf]

Additional figures

Figure S1. Distribution of the length of EST sequence (A) and length of contiged cluster of transcripts (B). Distribution of the size of EST clusters (C). This values correspond to the number of transcripts encoding for the same protein and are supposed to be related to gene expression level.

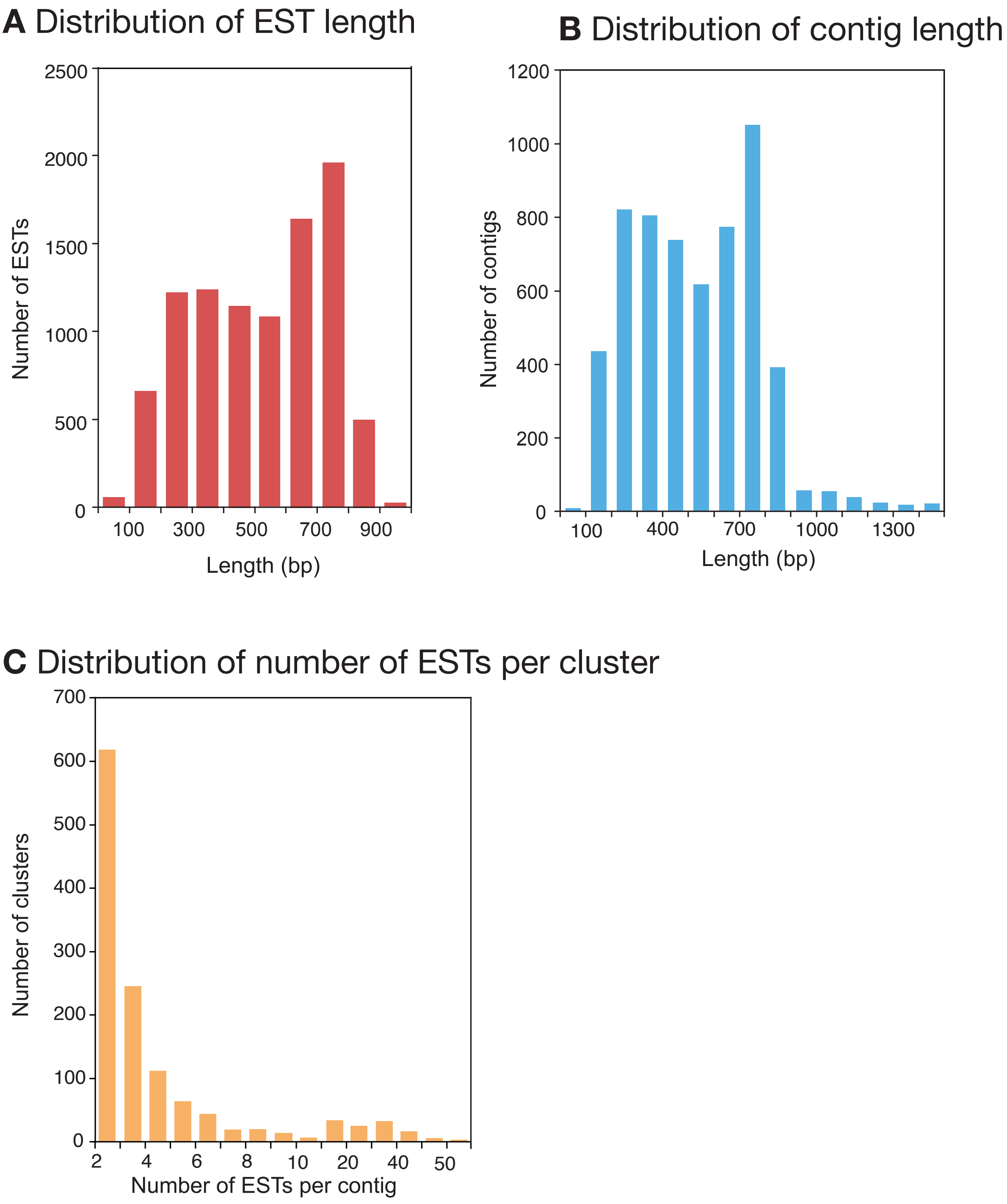

Figure S2. Summary of gene ontology annotation with plotting of the frequency of functional classes among transcript.

A Biological Process

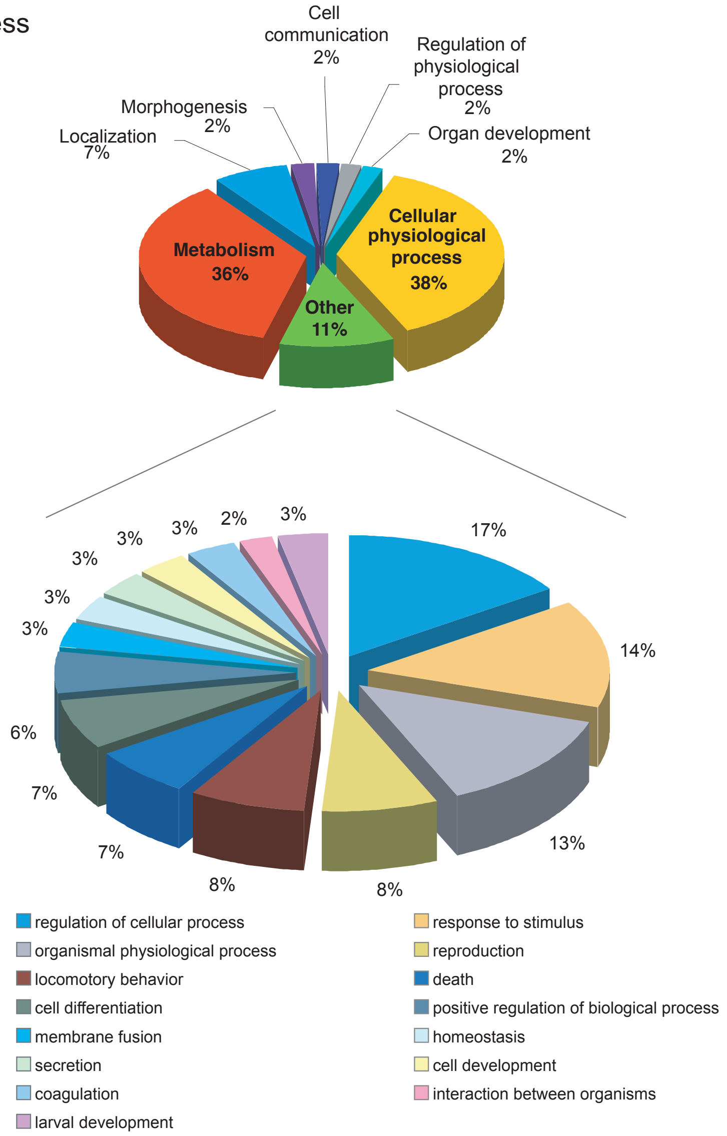

B Molecular Function

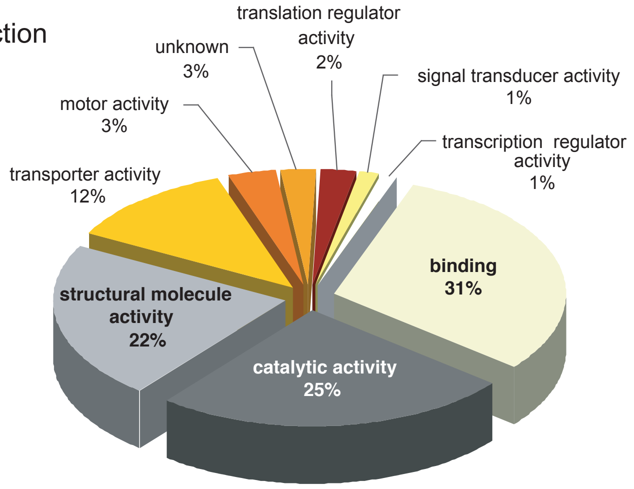

Figure S3. Alignment of Guanidinoacetate N-methyltransferase enzyme (GAMT) retrieved from selected taxa. This gene was not found in complete genomes of several lophotrochozoans (*Capitella capitata, Helobdella robusta, Lottia gigantea, Aplysia californica*, *Schmidtea mediterranea* and *Schistosoma mansoni)* nor in ecdysozoan protostomes (nematodes and insects) that are strongly sampled at the molecular level. The conserved positions are shown in red and the similar ones in blue.

Alignment of GMT sequences from representative organisms

```
Viridiplantae       1 WEKPLMEAHAKAICLNGGH------ILNVGFGMGLVDTAIQRYNPV-KHTIIEAHPEVYK
Fungi               1 WEDEIMQKSADLLVSRADKESDGPVVLNVGFGLGIIDTYLQSKKP-SKHYICEAHPDVLE
Porifera            1 WETPYMHKLATIAAGNGGR------VLEIGFGLAIAATKIQSHNNVSEHVIIECNEDVFH
Cnidaria            1 WETPYMHQLADIACSKGGR------VLEIGFGMAIAASRMETFK-LAEHVIVECNDEVFK
Acoela              1 WETPFMHELARVSMLNGGA------VLEVGFGMGISATGIQKYGP-TRHLIIEFNQAVID
Chaetognatha        1 WETPYMHSLARVARSHGGR------VLELGFGLAIAATEIEKG-NLEEHVIVECNEGVFK
Cephalochordata     1 WETPYMHKLAQVASCKGGR------VLEIGFGMAIAGSEIERQ-DIEEHWIIECNDGVFE
Craniata            1 WETPYMHSLSTVAASKGGR------VLEIGFGMAIAATKIESY-PIEEHWIIECNDGVFA
Urochordata         1 WETPYMHLLATIASSKGGK------VLEIGFGMAIAASKIQSR-AISEHWIIECNAGVFD
Hemichorda          1 WETPYMHALAKVASSKGGK------VLEIGFGMAIAATKIEEY-DIKEHVIIECNDGVFK
Echinodermata       1 WETPYMHELAKVASSKGGC------VLEIGFGLAIAATKIQEAPTVTEHVIIECNDGVFD

Viridiplantae      54 RMIESGWGEKENVKIVFGRWQDVLDKLDDN--SFDGIFFDTYGEYYED-----LREFHQH
Fungi              60 KMEKDGWMDKPGVTVLVGRWQDTLPGLLSQGVYFDGMYYDTFSENYSD-----LVDFFDH
Porifera           55 ELLKWAAESPRPVTPLQGLWQDVVPTLPDN--SFDGILYDTYPLSEETWHTHQFEFIKAH
Cnidaria           54 GLEKFAVDAPNKVTPLKGLWQDVIPTLEDG--SFDGIMYDTYPLSEDTWHTHQFEFIQKH
Acoela             54 KEGKSFAAAHPSVEILAGDWKDVMGSIADN--SLDGVLYDTYPMNKQEQHLHQFPFIQLA
Chaetognatha       54 RLEAWAKEQPNKVTPLKGLWEDVVHTLADN--SFDGILYDTYPLTEKEWHTHQFAFIVGH
Cephalochordata    54 RLEKWAKEQKHKIVPLKGMWQDVVATLPDG--HFDGILYDTYPLSEAEWHTHQFDFIGKH
Craniata           54 RLENWAKSQPHKVVPLKGLWENVVSTLPDN--HFDGILYDTYPLSEDTWHTHQFDFIKGH
Urochordata        54 RLQTWGKDQPHTVVPLKGMWEEVVPILPSG--HFDGILYDTYPLSEEDWHTHQFAFIKNH
Hemichorda         54 RLEEWAKKQPHKITPLKGMWEDVSPTLEDN--QFDGILYDTYPLSEQDWHTHQFNFIKQH
Echinodermata      55 RLEEWRKTQPHTVTPLKGMWEDVVPTLPDN--KFDGILYDTYPLSDATWHTHQFEFIKNH

Viridiplantae     107 LPRLLKPDGVYSYFNGFCGSNAFFHVVYCN-------------LV-TLEIENLGFSTQLI
Fungi             115 VVGLLAPTGVFSFFNGLGADRQVCYDVYKN----------VVEVDLQEYGLNVEYQVIKV
Porifera          113 AYRLLKPGGVLTYCNLTSWGELMKSSFDN--------IDKMFQETQVSHLIDAGFRRENI
Cnidaria          112 ASRLLKPGGVLTYCNLTSWGEFMKGKYDD--------IEKMFVETQIPKLIEAGFKRENI
Acoela            112 RPKLR-TGGVLTYCNLTSLGLLYSKYEKELGADKDKIWEAIWRETQVPNLLKAGWTADEL
Chaetognatha      112 AKRLLKPGGVLTYCNLTSWGEYMKKEFKGQ------SLNDMFESTQMPRLLDAGFKRENI
Cephalochordata   112 ACRLLKPGGVLSYCNLTSWGDLLKGKYDN--------IETMFKETQISHLEEAGFKKENI
Craniata          112 ANRLLKSGGVLTYCNLTSWGELLKTKYDN--------IEKMFEETQVPHLLQAGFKKEKI
Urochordata       112 AKRLLKPGGVLTYCNLTSWGELMKNKYTD--------IEQMFQETQVPXLVEAGFMKNKI
Hemichorda        112 AFRLLKPGGVLTYCNLTSWGELLKGKFDD--------IEKMFQETQVSHLEDAGFKKENI
Echinodermata     113 AFRLLKPGGVLTYCNLTSWGEFMKTKYTD--------IRQMFQETQVPSLCEAGFKKENI

Viridiplantae     153 PLPVKDCLGDEVWEGVKQKYWQLDTYYLPVCQFS-
Fungi             165 NKDVTGADG-HVWDGIKRRYWVVEDFYLPVCTF--
Porifera          165 KTEVMPI-----SPPSECRYYSYDKMIAPTITK--
Cnidaria          164 SWKVTDI-----VPEKECRYYSYSKMIAPTVVKS-
Acoela            171 AYHIFTLPQDAIEARGNCEYYSHATCLVPLLTKKR
Chaetognatha      166 STELMKI-----EPEPGCRYYEHEQMITPTIIKA-
Cephalochordata   164 SWECITN-----QPPKDCKYYQFPLMLAPKCIKA-
Craniata          164 STTTMDI-----APPTECKYYSFNKMITPTIVKD-
Urochordata       164 TTQVIDN-----KPPQDCKYYSTSKMIGPTIINE-
Hemichorda        164 STEVMDI-----EPEKECKYYSFRKMITPTIIK--
Echinodermata     165 STALLPI-----SPPKECRYYAFNHMITPTIIKA-
```

Figure S4. Neighbor-joining trees of selected strongly diverging genes from nuclear and mitochondrial genomes. (A) Tree from aminoacid alignment of RUX genes showing the ancient RUX-E and RUX-G that have both undergone subsequent duplication within the chaetognath lineage. (B,C) Relationships between the highly polymorphic sequences of mitochondrial genes Cytochrome Oxydase I (B) and III (C). Bootstrap support are indicated for selected nodes.

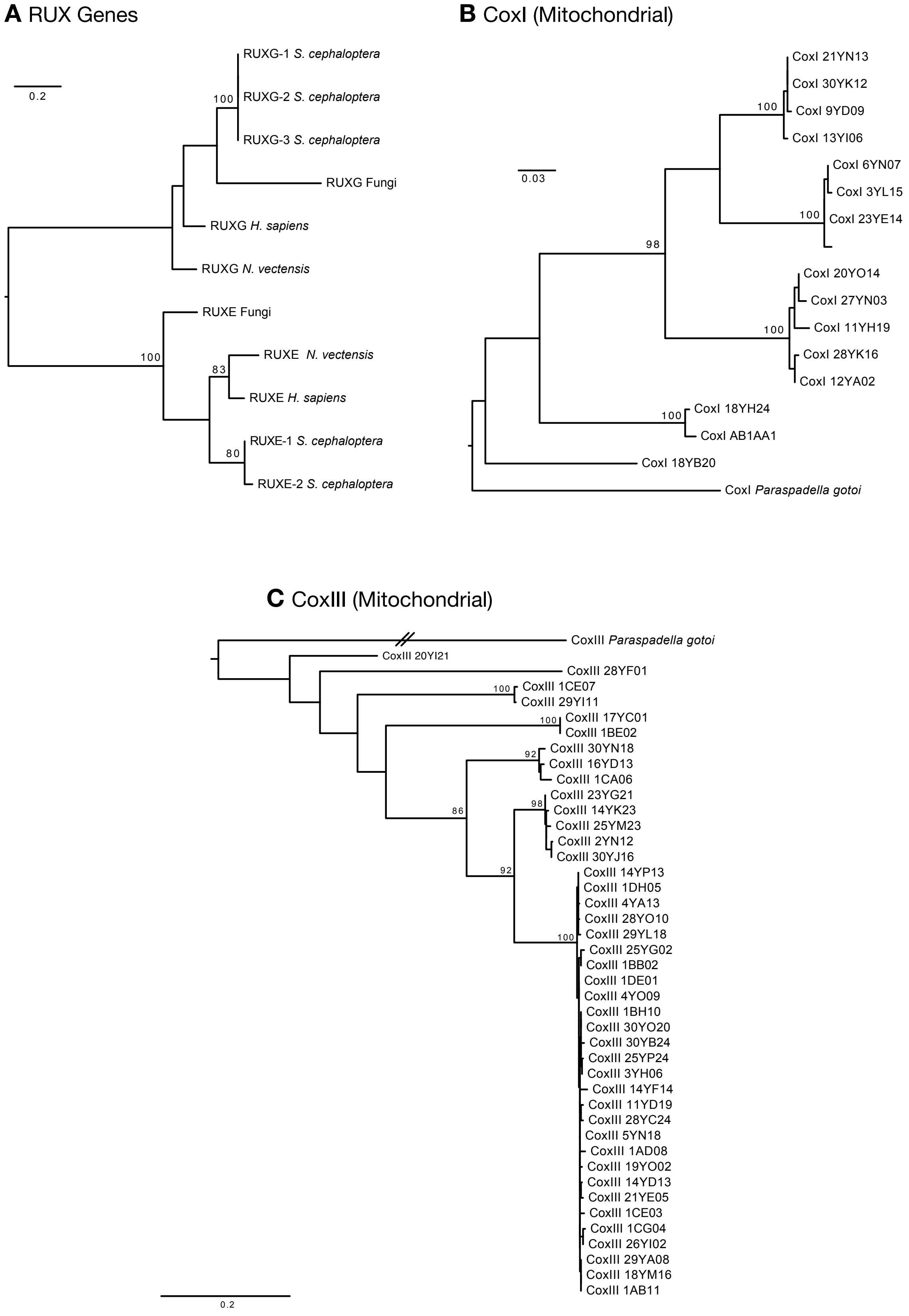

Figure S5. Transition versus transversion ratios for the 5 targeted genes. Pairwise divergence is computed assuming a kimura 2 parameters model.

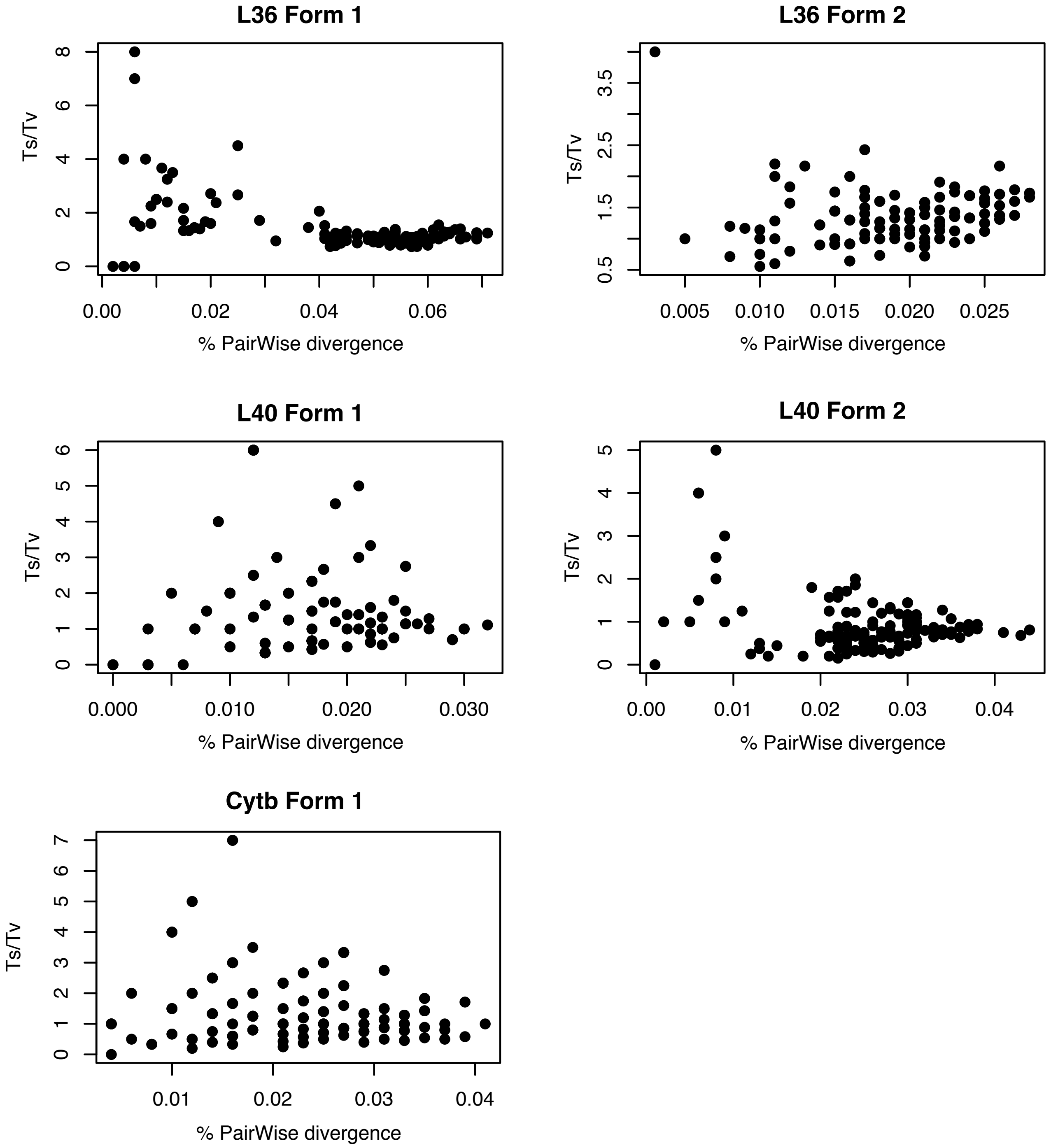

Figure S6. Mismatch analysis with distribution of pairwise divergence (case of cyt b, A) as well as Tajima and Fu & Li testing (B).

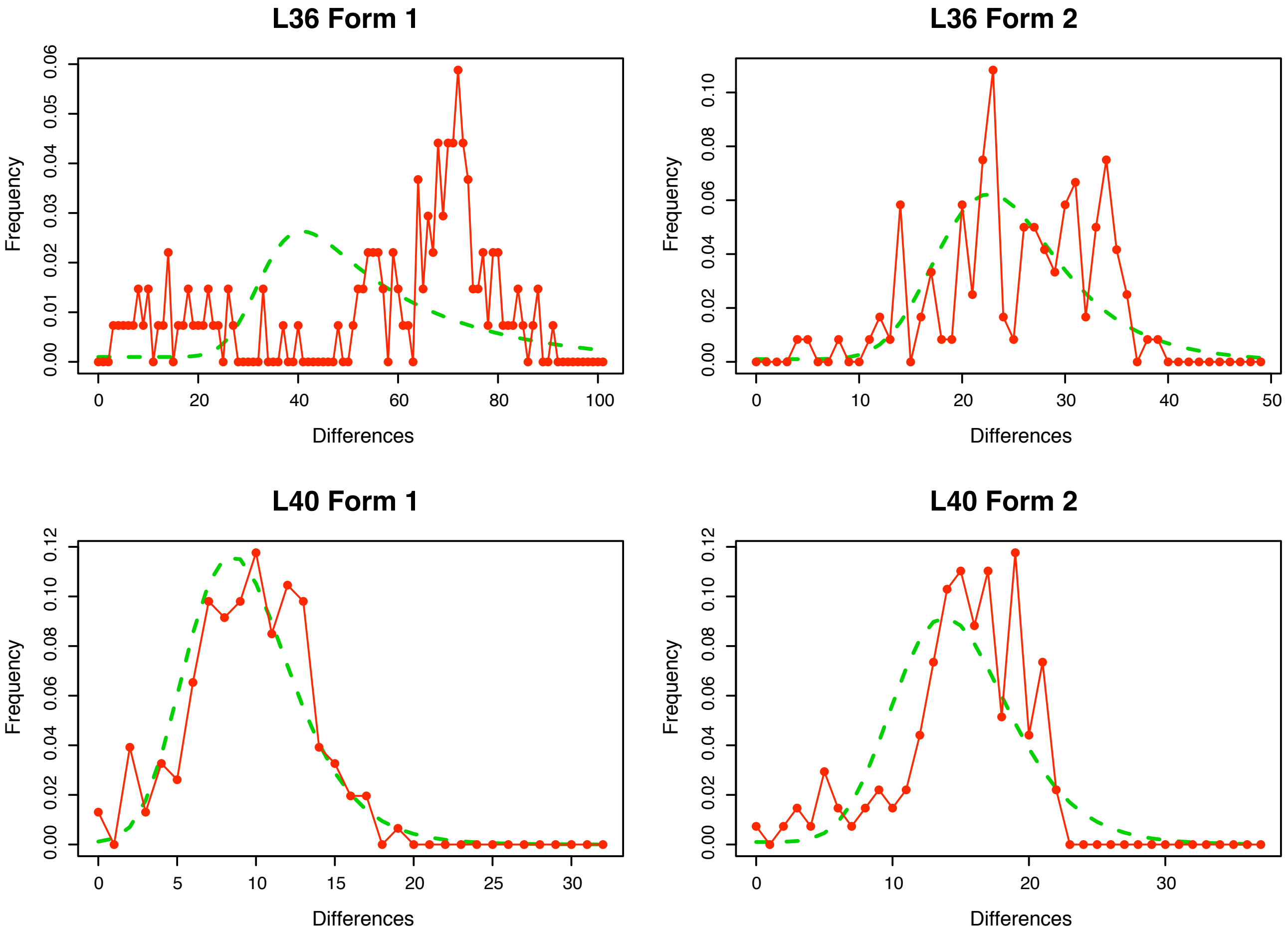

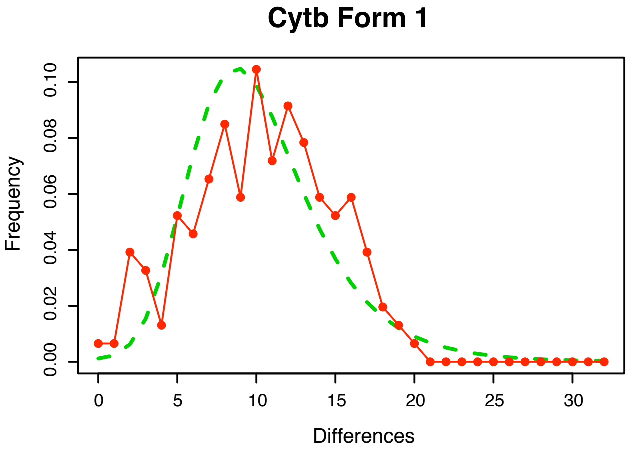

D testing results

Gene	Tajima's D	Fu and Li's D*	P-value
L40-1	-1,47454	-1,44136	P > 0.10
L40-2	-1,06718	-0,93174	P > 0.10
L36-1	-0,15000	-0,78526	P > 0.10
L36-2	-1,22504	-1,57929	P > 0.10
Cytb-1	-0,78547	-0,18120	P > 0.10
