# Supplementary material for: Chætognath transcriptome reveals ancestral and unique features among bilaterians
Source: Genome Biol. 2008 Jun 4;9(6):R94. doi: 10.1186/gb-2008-9-6-r94 (PMC2481426; doi:10.1186/gb-2008-9-6-r94)
Supplement: Additional data file 2 — Table S1: composition of the composite taxa involved in phylogenomic analyses. Table S2: list of all clusters of transcripts corresponding to alternative forms or not with Ka/Ks ratios. Table S3: list of primers employed for PCR amplifications of alternative forms. Table S4: comparison of molecular evolution trends for four genes retrieved in nine individuals. Table S5: annotation of the BAC 35A21. [file gb-2008-9-6-r94-S2.pdf]

# Additional tables

**Table S1** Composition of the composite taxa employed in phylogenomic analysis. The amount of missing data was determined for the final Gblocked alignment of 11 730 aminoacid positions. The database size correspond to the amount of EST available for each taxon (see methods). The number of species included in the taxon as well as the name of principal species are specified.

| Taxon | Missing data | Database size | Number of Species | Principal species* |
|---|---|---|---|---|
| **Fungi** | 0.2% | 1 590 310 | 26 | ***Blastocladiella emersonii***, *Aspergillus flavus, Glomus intraradices, Cryphonectria parasitica, Phanerochaete chrysosporium, Magnaporthe grisea* |
| **Choanoflagellata** | 2.3% | 7 528 | 1 | ***Monosiga ovata*** |
| **Homoscleromorpha** | 46.1% | 11 186 | 1 | ***Oscarella carmella*** |
| **Desmospongia** | 0.0% | 83 040 | 2 | ***Amphimedon queenslandica,*** *Suberites domuncula* |
| **Placozoa** | 2.3 % | WGS | | ***Trichoplax adherens*** |
| **Ctenophora** | 11.3 % | 15 752 | 1 | ***Mnemiopsis leidyi*** |
| **Anthozoa** | 2.8% | 39 428 | 2 | ***Nematostella vectensis,*** *Acropora millepora* |
| **Hydrozoa** | 0.0% | 198 739 | 3 | ***Hydra magnipapillata***, *Hydractinia echinata, Podocoryne carnea* |
| **Craniata** | 0.0% | 2 102 016 | 16 | ***Homo sapiens***, *Danio rerio, Ictalurus punctatus, Xenopus laevis, Mus musculus* |
| **Urochordata** | 0.4% | 894 642 | 1 | ***Ciona intestinalis*** |
| **Cephalochordata** | 0.0% | 262 571 | 1 | ***Branchiostoma floridae*** |
| **Echinodermata** | 0.2% | 824 466 | 5 | ***Strongylocentrotus purpuratus***, *Paracentrotus lividus, Asterina pectinifera, Hemicentrotus pulcherrimus, Heliocidaris erythrogramma* |
| **Hemichordata** | 0.5% | 256 000 | 1 | ***Saccoglossus kowalevskii*** |
| **Xenoturbellida** | 38.8 % | 2 137 | 1 | ***Xenoturbella bocki*** |
| **Chaetognatha** | 6.0% | 11 526 | 2 | ***Spadella cephaloptera***, *Flaccisagitta enflata* |
| **Tardigrada** | 23.0% | 5 235 | 1 | ***Hypsibius dujardini*** |
| **Nematoda** | 0.6% | 275 019 | 25 | ***Ascaris suum***, *Litomosoides sigmodontis, Pristionchus pacificus, Meloidogyne incognita, Haemonchus contortus, Trichinella spiralis* |
| **Priapulida** | 56.4% | 478 | 1 | ***Priapulus caudatus*** |
| **Insecta** | 0.0% | 1 906 211 | 21 | ***Spodoptera frugiperda***, *Nasonia vitripennis, Aedes aegypti, Bombyx mori, Drosophila willistoni* |
| **Crustacea** | 0.6% | 109 254 | 13 | ***Litopenaeus vannamei,*** *Penaeus monodon, Callinectes sapidus, Marsupenaeus japonicus, Homarus americanus* |
| **Chelicerata** | 0.0% | 117 871 | 12 | ***Rhipicephalus appendiculatus***, *Boophilus microplus, Mesobuthus gibbosus, Amblyomma americanum, Cupiennius salei, Ornithodoros porcinus* |
| **Onychophora** | 70.1 % | 1 868 | 1 | ***Epiperipatus sp.*** |
| **Mollusca** | 1.3% | 326 977 | 16 | ***Argopecten irradians ,*** *Chlamys farreri, Aplysia californica, Crassostrea virginica, Crassostrea gigas* |
| **Annelida** | 1.0% | 27 297 | 2 | ***Lumbricus rubellus ,*** *Platynereis dumerilii* |
| **Nemertea** | 24.2 % | n.d. | 1 | ***Lineus viridis*** |
| **Ectoprocta** | 12.8 % | 4 074 | 1 | ***Flustra foliacea*** |
| **Entoprocta** | 45.0 % | 2 154 | 1 | ***Barentsia elongata*** |
| **Platyhelminthes** | 0.1% | 385 113 | 11 | ***Macrostomum lignano,*** *Echinococcus granulosus, Schistosoma japonicum, Schistosoma mansoni, Schmidtea mediterranea, Dugesia japonica, Clonorchis sinensis* |
| **Rotifera** | 28.3 % | 290+412** | n.d. | **Rotifera sequences extracted from Oryza sativa EST libraries,** *Philodina roseola* |

* 5 or 6 more abundant are mentioned, the most abundant species is in bold type

** correspond to the number of ESTs extracted from rice EST database using the SL as an anchor

n.d. = not determined

**Table S2**. Detailed list of all clusters including more than 6 similar transcripts or clearly displaying alternative forms. For duplicated genes, Ka/Ks is precised.

**Ribosomal protein genes**

| Name | #Duplicates | #ESTs | Ka/Ks | Ks | Description |
|---|---|---|---|---|---|
| RP S8 | 3 | 23 | 0.003 to 0.08 | 1.98 to 3.18 | 40S Ribosomal Protein S8 |
| RP L36 | 2 | 49 | 0.0015 | 27.9328 | 60S Ribosomal Protein L36 |
| RP L40 | 2 | 43 | 0.0027 | 6.9742 | 40S Ribosomal Protein L40 |
| RP L27 | 2 | 42 | 0.0353 | 1.5075 | 60S Ribosomal Protein L27 |
| RP L44 | 2 | 42 | 0.0085 | 1.9813 | 60S Ribosomal Protein L44 |
| RP S27 | 2 | 40 | 0.0119 | 1.7791 | 40S Ribosomal Protein S27 |
| RP L38 | 2 | 37 | 0.0141 | 3.1357 | 60S Ribosomal Protein L38 |
| RP L30 | 2 | 36 | 0.001 | 29.3261 | 60S Ribosomal Protein L30 |
| RP L7 | 2 | 35 | 0.0249 | 3.4038 | 60S Ribosomal Protein L7 |
| RP S17 | 2 | 30 | 0.0022 | 38.3237 | 40S Ribosomal Protein S17 |
| RP L35 | 2 | 27 | 0.001 | 75.2599 | 60S Ribosomal Protein L35 |
| RP S16 | 2 | 26 | 0.0016 | 11.4501 | 40S Ribosomal Protein S16 |
| RP L34 | 2 | 24 | 0.0193 | 1.7984 | 60S Ribosomal Protein L34 |
| RP S23 | 2 | 24 | 0.001 | 2.0549 | 40S Ribosomal Protein S23 |
| RP L22 | 2 | 23 | 0.001 | 44.1484 | 60S Ribosomal Protein L22 |
| RP S28 | 2 | 23 | 0.1539 | 1.2698 | 40S Ribosomal Protein S28 |
| RP S1A | 2 | 21 | 0.001 | 2.5249 | 40S Ribosomal Protein S1A |
| RP S25 | 2 | 17 | 0.025 | 3.9945 | 40S Ribosomal Protein S25 |
| RP S15 | 2 | 16 | 0.001 | 0.9422 | 40S Ribosomal Protein S15 |
| RP S13 | 2 | 14 | 0.0017 | 1.741 | 40S Ribosomal Protein S13 |
| RP S24 | 2 | 14 | 0.0012 | 2.9587 | 40S Ribosomal Protein S24 |
| RP S21 | 2 | 13 | 0.023 | 3.9136 | 40S Ribosomal Protein S21 |
| RP S27A | 2 | 12 | 0.0013 | 7.9757 | 40S Ribosomal Protein S27a |
| RP L13A | 2 | 12 | 0.0421 | 1.5282 | 60S Ribosomal Protein L13A |
| RP L2A | 2 | 12 | 0.0274 | 1.5525 | 60S Ribosomal Protein L2A |
| RP L32 | 2 | 11 | 0.001 | 1.9579 | 60S Ribosomal Protein L32 |
| RP L14 | 2 | 9 | 0.001 | 0.8011 | 60S Ribosomal Protein L14 |
| RP L21 | 2 | 8 | 0.0175 | 3.6456 | 60S Ribosomal Protein L21 |
| RP S4 | 2 | 6 | 0.0162 | 1.8372 | 40S Ribosomal Protein S4 |
| RP L15 | 2 | 2 | 0.001 | 10.6474 | 60S Ribosomal Protein L15 |
| RP S9 | 1 | 45 | - | - | 40S Ribosomal Protein S9 |
| RP S29 | 1 | 37 | - | - | 40S Ribosomal Protein S29 |
| RP S7 | 1 | 31 | - | - | 40S Ribosomal Protein S7 |
| RP L10 | 1 | 31 | - | - | 60S Ribosomal Protein L10 |
| RP S19 | 1 | 30 | - | - | 40S Ribosomal Protein S19 |
| RP S11 | 1 | 27 | - | - | 40S Ribosomal Protein S11 |
| RP L6 | 1 | 20 | - | - | 60S Ribosomal Protein L6 |
| RP L1X | 1 | 17 | - | - | 60S Ribosomal Protein L1X |
| RP L29 | 1 | 12 | - | - | 60S Ribosomal Protein L29 |
| RP L31 | 1 | 11 | - | - | 60S Ribosomal Protein L31 |
| RP L37 | 1 | 10 | - | - | 60S Ribosomal Protein L37 |
| RP L37A | 1 | 9 | - | - | 60S Ribosomal Protein L37A |
| RP L28 | 1 | 7 | - | - | 60S Ribosomal Protein L28 |
| RP S14 | 1 | 7 | - | - | 40S Ribosomal Protein S14 |
| RP L11 | 1 | 6 | - | - | 60S Ribosomal Protein L11 |
| RP L23 | 1 | 6 | - | - | 60S Ribosomal Protein L23 |
| RP LA1 | 1 | 6 | - | - | 60S Ribosomal Protein LA1 |

**Table S2** (continued).

## Other Genes

| Name | #Duplicates | #ESTs | Ka/Ks | Ks | Description |
|---|---|---|---|---|---|
| H33 | 3 | 23 | 0.001 | 1.55 to 2.05 | Histone H3.3 |
| RUXG | 3 | 16 | 0.0266 | 2.0481 | Small nuclear ribonucleoprotein G |
| MLE | 3 | 7 | 0.002 to 0.004 | 59.7 to 64.1 | Myosin essential light chain |
| RUXE | 2 | 10 | 0.0059 | 1.4941 | Small nuclear ribonucleoprotein E |
| PDCD6 | 2 | 6 | 0.052 | 4.872 | Programmed cell death protein 6 |
| SUMO | 2 | 6 | 0.0102 | 5.2782 | Small ubiquitin-related modifier precuRP Sc |
| TCTP | 1 | 112 | - | - | Translationally-controlled tumor protein |
| PPIA | 1 | 25 | - | - | Peptidyl-prolyl cis-trans isomerase |
| ATPK | 1 | 21 | - | - | ATP synthase f chain |
| H2B | 1 | 15 | - | - | Histone H2B |
| CALM | 1 | 11 | | - | Calmodulin |
| EF1D | 1 | 10 | - | - | Elongation factor 1 delta |
| SC61G | 1 | 10 | - | - | Transport protein SEC61 subunit gamma |
| H2AX | 1 | 7 | - | - | Histone H2A.x |
| ACPM | 1 | 6 | - | - | Acyl carrier protein. mitochondrial precuRP |
| FABPB | 1 | 6 | - | - | Fatty acid-binding protein |
| H2AL | 1 | 6 | - | - | Late histone H2A.L3 |
| HINT1 | 1 | 6 | - | - | Histidine triad nucleotide-binding protein 1 |
| NH2L1 | 1 | 6 | - | - | Nuclear high mobility group protein 2 |
| SMD2 | 1 | 6 | - | - | Small nuclear ribonucleoprotein Sm D2 |

**Table S3.** PCR primers designed to perform the amplification of alternative forms (paralogous copies) of targeted genes, RP L36 and L40 and Cyt b. The position of primers is given from the start of CDS and always refered to maximal length product in case of length polymorphism. In case introns were present in the product, the position of the primer on the transcript is also specified in brackets for some cases.

| Name | Sequence (5'-3') | Position | Product length |
|---|---|---|---|
| Cytb 1 For | CTC GTC TTC CTA AAC GGG GC | 11 | 487 |
| Cytb 1 Rev | CAC AGC CAC ACC ACA AAA TCG | 497 | |
| Cytb 2 For | TAC CGC AAC TTG GAC TGT AGG G | 330 | 175 |
| Cytb 2 Rev | ACC ACC TCA TAG CCA TAC CAC CAG | 504 | |
| Cytb 3 For | CCT GAG GGC AGA TAA GGT TTT GG | 404 | 133 |
| Cytb 3 Rev | AAG CGG GTG AGC GTA GCG TTT C | 536 | |
| L36 Generic | TGG CTC CCA AAT ACG CTA TGG | 2 | - |
| L36 1 Rev | CAA CCT TAC TGA AGC GAT TGA AAA C | 1427 (520) | 1425 |
| L36 2 For | CGA AGG AAC TGG AAG AGA CAA AAG | 1488 (521) | 1485 |
| L40 1 For | TGG AAG ATG GTC GCA CTC TCT CTG | 148 | 889 |
| L40 1 Rev | ACG CCT CGT GAC AAC AAG TTA TG | 1038 (414) | |
| L40 2 For | TGC GTT TGC GTG GAG GTA TC | 211 | 894 |
| L40 2 Rev | TTG GGA GGA AGG TTG TTG GTG TGC | 1106 (347) | |

**Table S4.** Comparisons of sequences from alternative sequenced marker genes for 9 individuals. Various molecular evolution and sequence parameters are shown.

| Gene | L40 Form 1 | L40 Form 2 | L36 Form 1 | L36 Form 2 |
|---|---|---|---|---|
| Sequence Length | 889 | 894 | 1426 | 1488 |
| #variable sites | 55 | 93 | 191 | 126 |
| #Singletons | 29 | 49 | 84 | 76 |
| #Transitions | 23 | 26 | 62 | 47 |
| #AG | 11 | 10 | 36 | 22 |
| #CT | 12 | 16 | 26 | 25 |
| AT rich | 62% | 65% | 59.5% | 61.3% |
| K2P distance | 0.003 to 0.033 | 0.001 to 0.046 | 0.002 to 0.075 | 0.003 to 0.028 |

**Table S5.** Detailed composition of BAC 35A21. Annotations include both the coding sequences predicted by Augustus that have a significant match against SwissProt and the ESTs mapped onto Blast sequence by Blast.

**Protein coding genes** similar to SwissProt sequences

| # | Start | End | Orientation | Score | Similar to |
|---|---|---|---|---|---|
| g1 | 99 | 1203 | + | 409 | GDP dissociation inhibitor 1 |
| g2 | 2072 | 3292 | + | 231 | 40S ribosomal protein S14 |
| g3 | 3584 | 6253 | + | 233 | Proliferating cell nuclear antigen (PCNA) |
| g5 | 9655 | 13601 | - | 759 | Gag-polyprotein, transposon |
| g6 | 16627 | 22509 | + | 172 | Thioredoxin domain containing 13 |
| g7 | 23064 | 23906 | + | 186 | RING finger protein 170 |
| g8/9 | 24720 | 39722 | + | 176 | Sorting Nexin 17 |
| g11 | 44307 | 46234 | + | 90 | Tyrosine recombinase |
| g12 | 48192 | 51098 | + | 326 | COP9 complex subunit 2 |
| g13 | 65484 | 67785 | + | 56 | Zinc finger protein 513 |
| g15 | 69222 | 70895 | + | 356 | Chondroitin synthase |
| g16 | 71376 | 72272 | + | 196 | Chsy1 protein |
| g17 | 74697 | 75543 | + | 51 | Transposon proteins |
| g19 | 75913 | 76979 | + | 233 | |
| g20 | 77036 | 80201 | + | 186 | |
| g26 | 90147 | 96999 | + | 207 | protein regulator of cytokinesis 1 |
| g27 | 97657 | 98739 | | | unidentified protein |
| g28 | 10187 | 101428 | + | 140 | NADH dehydrogenase 1 alpha subcomplex |
| g29 | 101628 | 103749 | + | 188 | Elongation factor Ts, mitochondrial precursor |
| g31 | 106583 | 107770 | - | 244 | Transposon proteins |
| g31 | 107889 | 109259 | - | 184 | |
| g35 | 116352 | 119826 | + | 86 | death-associated small cytoplasmic leucine-rich protein SCLP |
| g40 | 134869 | 140691 | - | 108 | Transposon proteins |
| g41 | 143853 | 157798 | + | 551 | |

**Mapped ESTs** similar to coding sequences

| EST ID | Start | End | Similarity | Score* | Related gene |
|---|---|---|---|---|---|
| ADA26YF03 | 2403 | 3360 | 97.64 | 199 | 40S ribosomal protein S14 |
| ADA16YG13 | 2403 | 3494 | 97.64 | 309 | |
| ADA18YA01 | 2403 | 3494 | 99.28 | 307 | |
| ADA21YB19 | 2403 | 3491 | 98.37 | 309 | |
| ADA6YI24 | 2403 | 3393 | 96.94 | 243 | |
| ADA25YC10 | 2403 | 3393 | 98.53 | 243 | |
| ADA24YH20 | 3101 | 3494 | 96.94 | 306 | |
| ADA1CC08 | 3477 | 5209 | 100.00 | 281 | Proliferating cell nuclear antigen (PCNA) |
| ADA18YB09 | 48755 | 49255 | 98.39 | 399 | COP9 complex subunit 2 |
| ADA6YE11 | 48755 | 49174 | 97.64 | 333 | |
| ADA6YJ13 | 98161 | 98739 | 91.78 | 161 | NADH dehydrogenase (ubiquinone) 1 alpha subcomplex |

** blastn score against BAC DNA sequence*
